# Supplementary material for: Disulfide isomerization reactions in titin immunoglobulin domains enable a mode of protein elasticity
Source: Nat Commun. 2018 Jan 12;9:185. doi: 10.1038/s41467-017-02528-7 (PMC5766482; doi:10.1038/s41467-017-02528-7)
Supplement: Supplementary file 1 — Supplementary Information [file 41467_2017_2528_MOESM1_ESM.pdf]

Supplementary Figures

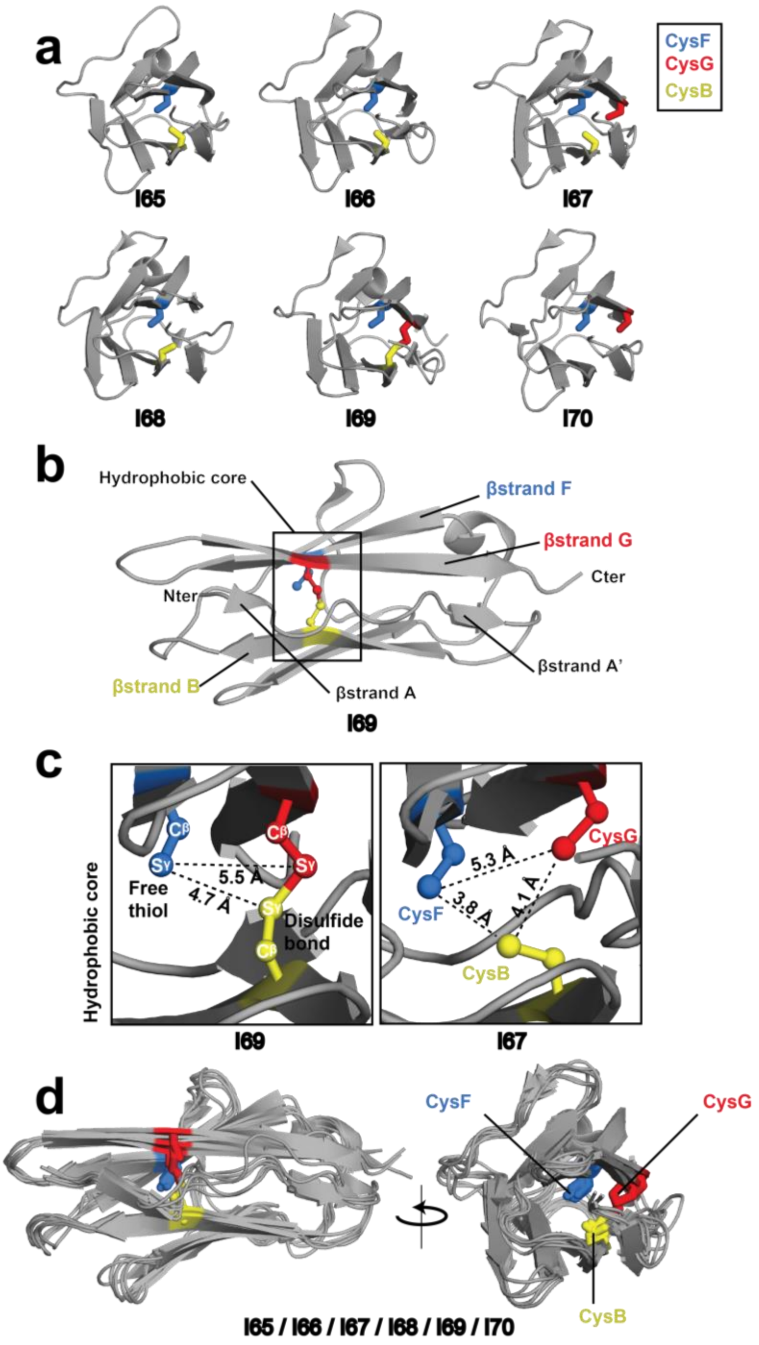

Supplementary Figure 1 – Cysteines in the I65-I70 hexamer

(a) The Xray structures of the 6 domains from the hexamer I65-I70 (pdb:3B43) are shown. Cysteines are represented in colored licorice. **(b)** The structure of I69 reveals disulfide CysB-CysG, while thiol of CysF remains free and close to the CysB-CysG bond. **(c)** CysB, CysF and CysG are co-localized in the hydrophobic core of I67 and I69. **(d)** Superimposed domains show a conservation of the overall structure, orientation of cysteine side chains and conformation of the mechanical clamp motif.

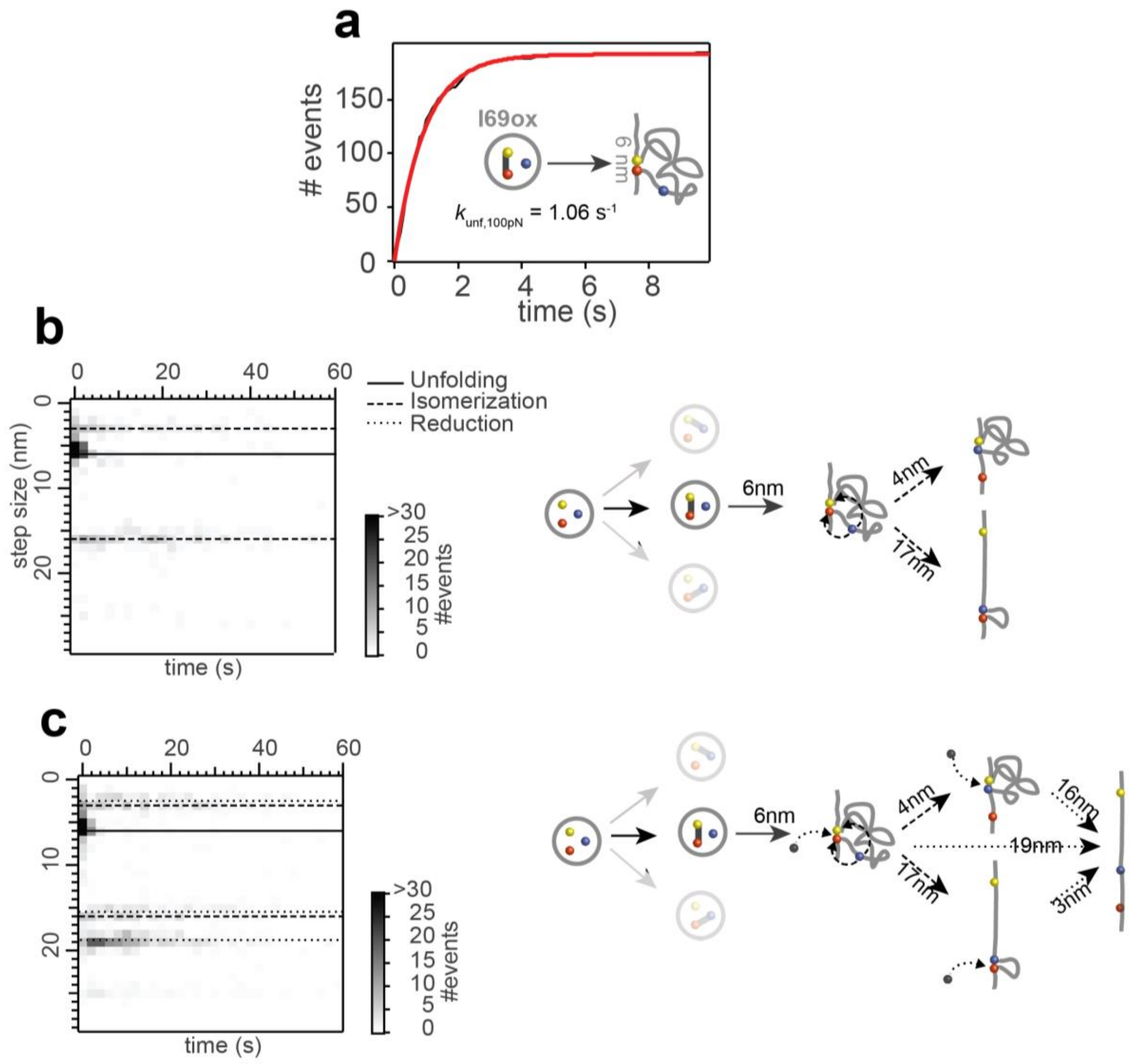

Supplementary Figure 2 – Dwell-time distribution of unfolding, isomerization and reduction of I69

(a) *Black curve corresponds to* the time course of unfolding for $(I69)_8$ represented as cumulative histograms obtained from several individual recordings in the oxidized state (6 nm steps) at 100 pN. Exponential fit is shown with a red curve. **(b)** Density plot of the time course of the different step sizes recorded while $(I69_{oxidized})_{8,}$ is extended at 100 pN. **(c)** Density plot of the time course of the different step populations recorded while $(I69_{oxidized})_8$ extends at 100 pN in the presence of reducing agent (10mM Tcep). Schemes on the right illustrate the sequence of step sizes in these experiments. The only disulfide bond observed in $I69_{oxidized}$ is CysB-CysG . This disulfide bond is cryptic and inaccessible to Tcep in the folded state; however, unfolding exposes the disulfide to solution and enables redox reactions.

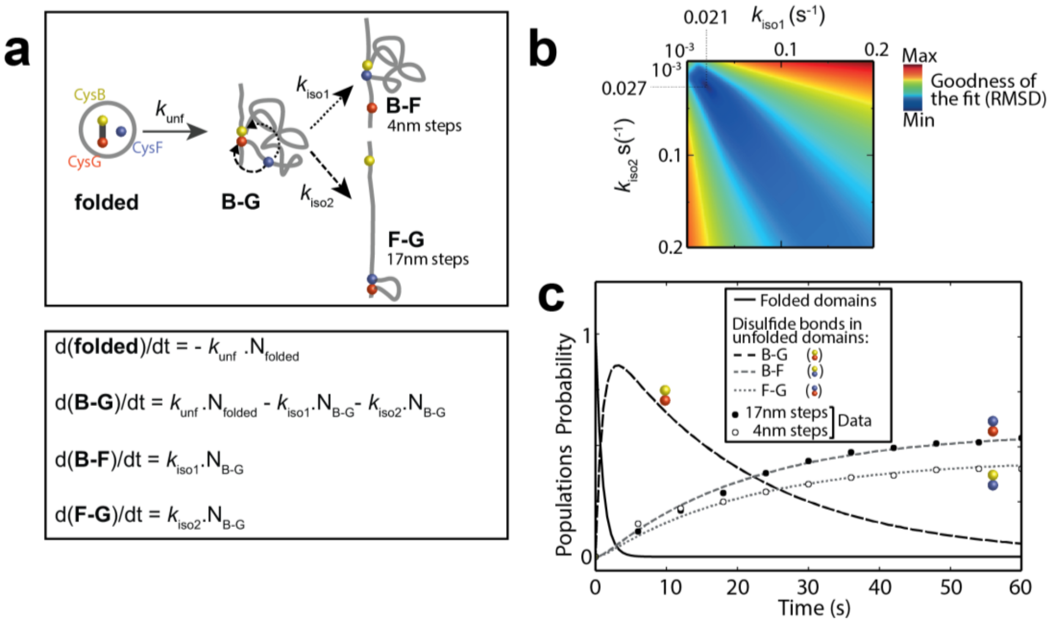

Supplementary Figure 3 – Kinetics of disulfide isomerization in I69

(a) We investigate the kinetics of $I69_{oxidized}$ unfolding and subsequent disulfide isomerizations under a constant force of 100 pN (Figure 3b,c). $I69_{oxidized}$ extends with 3 distinct populations of steps corresponding to unfolding events (6 nm) or the two possible events of disulfide bond isomerization (4 and 17 nm). The transition rates (i.e. rate constants k_{unf}, k_{iso1} and k_{iso2}) between the four states (folded or unfolded with the disulfides CysB-CysG, CysB-CysF and CysF-CysG) can be integrated in a system governed by coupled differential equations (lower panel). In this model, we consider that the isomerization of the disulfide CysB-CysG can occur only in the unfolded state. The analytical solution to the system shows the probability of the system being in one of the four states as a function of time. We consider that the modeled system is in the folded state at the beginning of the experiment. **(b)** To estimate rate constants, we compare the evolution of the system with the experimental data obtained with traces longer than 20 seconds, which ensures that we are capturing most of the isomerization events [5,16,17]. Indeed, we observe that less than 10% of isomerization events are missing from experimental traces containing a total of 202 6 nm unfolding events. Experimental data is computed from the dwell time of each particular event summed in cumulative histograms. First, k_{unf} is estimated independently by fitting a single exponential to the histogram of the appearance of 6 nm steps (k_{unf} = 1.06s^{-1}, Supplementary Fig. 2a). We then find the optimal parameter values k_{iso1} and k_{iso2} that best fit our data and describe the appearance of the 4 nm and 17 nm steps in our experimental recordings. For a given set of rate constants, we evaluate the goodness of the fit by calculating root mean square deviations (RMSD). We use the Downhill simplex algorithm (Scipy-Python2.7) which converges to a global minimal $RMSD_{minimum}$ = 2.22 associated to the rate constants k_{iso1} = 0.027 s^{-1} and k_{iso2} =0.021 s^{-1}. To illustrate this result and confirm the presence of one single solution to solve the system, we plot RMSD resulting from varying k_{iso1} and k_{iso2} between 0,001 and 0.200 s^{-1} with an increment of 0.001. The resulting 2D plot confirms the presence of one simple global minimum that matches the calculated parameters k_{iso1} and k_{iso2}. **(c)** Evolution of the system over time with the calculated parameters that fit the experimental data (circles).

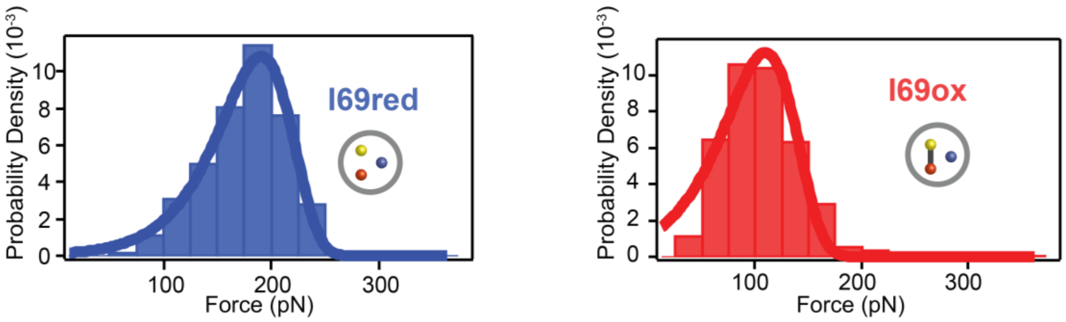

Supplementary Figure 4 – Mechanical unfolding parameters of $I69_{reduced}$ and $I69_{oxidized}$
To estimate the change of unfolding probability of I69 at different stretching forces, we calculate the rate of unfolding at zero force (α_0) and the distance to the transition state (Δx) from force-ramp experiments in which force increases at a constant rate of 40 pN·s^{-1} [18]. From multiple traces, we assess the force at which unfolding events occur. We report the probability distribution of unfolding forces for $I69_{reduced}$ (blue) and $I69_{oxidized}$ (red). We obtain α_0 = 0.0044 s^{-1} and Δx = 0.12 nm for $I69_{reduced}$ and α_0 = 0.0466 s^{-1} and Δx = 0.12 nm for $I69_{oxidized}$. When the disulfide bond CysB-CysG is formed in I69, the distance to the transition state Δx remains unaffected whereas the unfolding rate extrapolated to zero force, α_0, is 10 times higher.

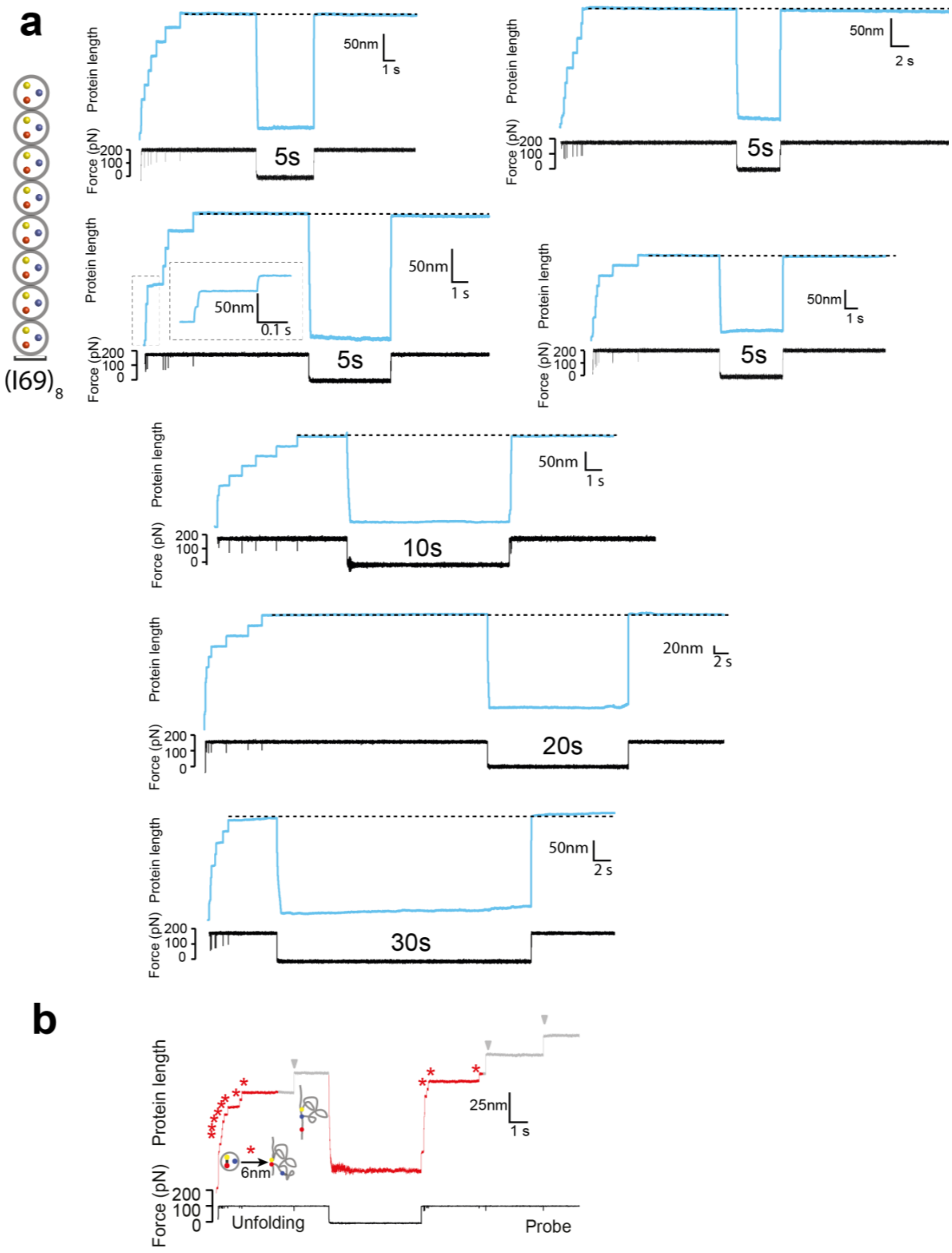

Supplementary Figure 5 – AFM experiments show the low ability of I69$_{reduced}$ to refold
(a) In these experimental traces, we evaluate the refolding ability of I69$_{reduced}$ in absence of disulfide bonds [19]. Here, we use a typical force protocol divided in three pulses (unfolding-quench-probe). We probe the ratio of domains that regain their native state during the quench (0pN for a set time *Δt*) after being mechanically unfolded. The first unfolding pulse applied to (I69)$_8$ produces a typical staircase of 26 nm steps. We were not able to detect refolded domains unfolding in the probe pulse even for quench times of 30 s. Dotted lines verify that the final extension in the first pulse and the probe pulse are the same. **(b)** When homopolyproteins are used in AFM experiments, the impact of disulfide bonds in the folding of I69$_{oxidized}$ domains remains elusive because of isomerization reactions that make results difficult to interpret. In this particular trace, a heterogeneous population of disulfide bonds is present at the end of the unfolding pulse (7 CysB-

CysG and 1 additional CysF-CysG that formed by isomerization, grey triangle). The three 6 nm steps observed in the probe pulse suggest that domains harboring the CysB-CysG bond are able to refold. However, in this trace it is challenging to verify that a single-molecule tether remains after the quench since multiple isomerization reactions change the total contour length of the protein.

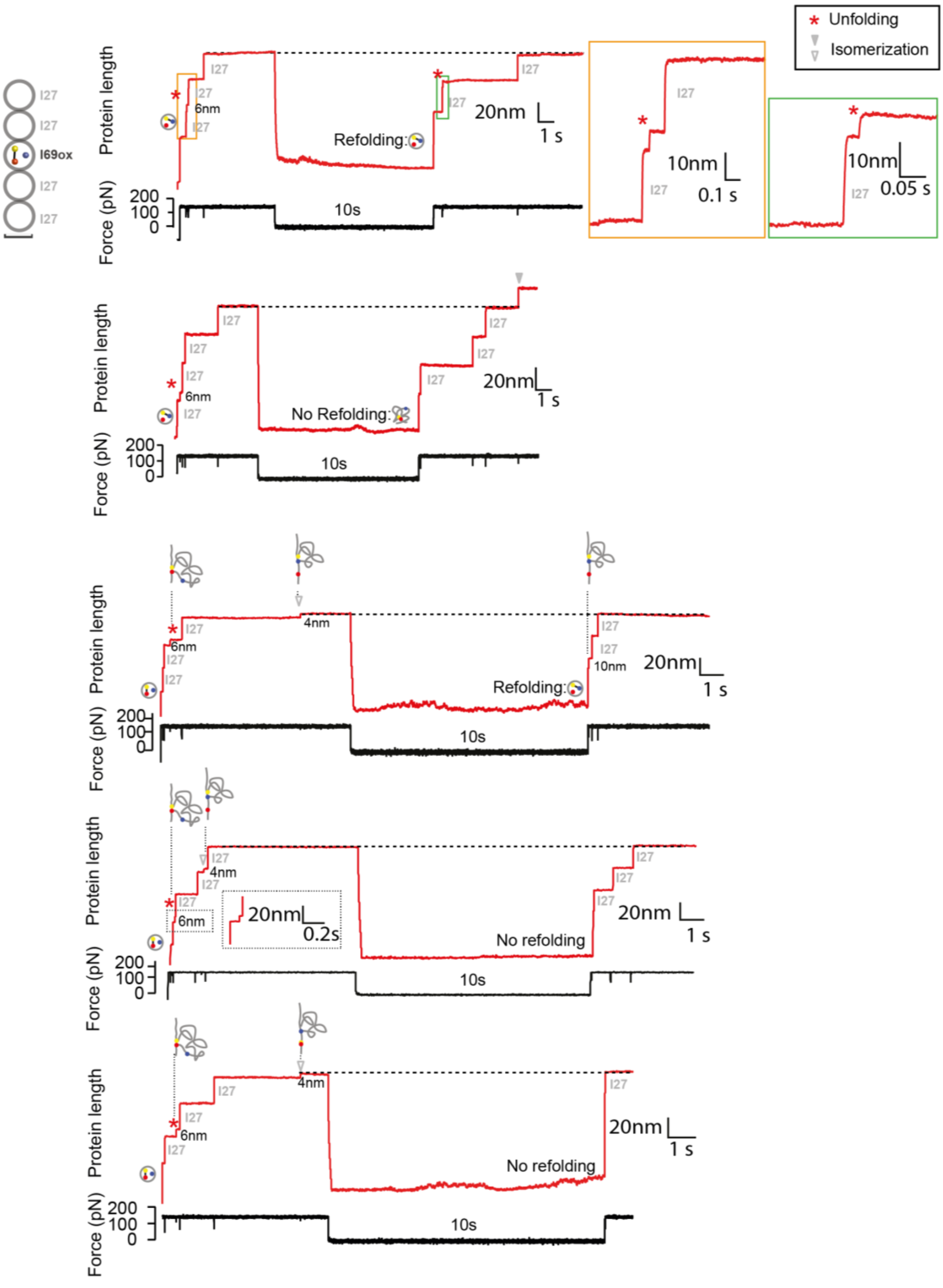

Supplementary Figure 6 – Efficient refolding of $I69_{oxidized}$ in the construct $(I91\Delta Cys)_2$-$I69_{oxidized}$-$(I91\ \Delta Cys)_2$

In this figure, we present five additional recordings in which the polyprotein $(I91\Delta Cys)_2$-$I69_{oxidized}$-$(I91\Delta Cys)_2$ extends at constant force (see also Figure 5 in the main text). We track in these traces the disulfide bond exchange in the first pulse (4nm or 17nm steps). Consequently, we can monitor how each disulfide affects the folding ability. A shorter step of 6 nm, 10 nm or 23 nm in the probe pulse marks the unfolding of $I69_{oxidized}$ with its three alternative disulfide bonds.

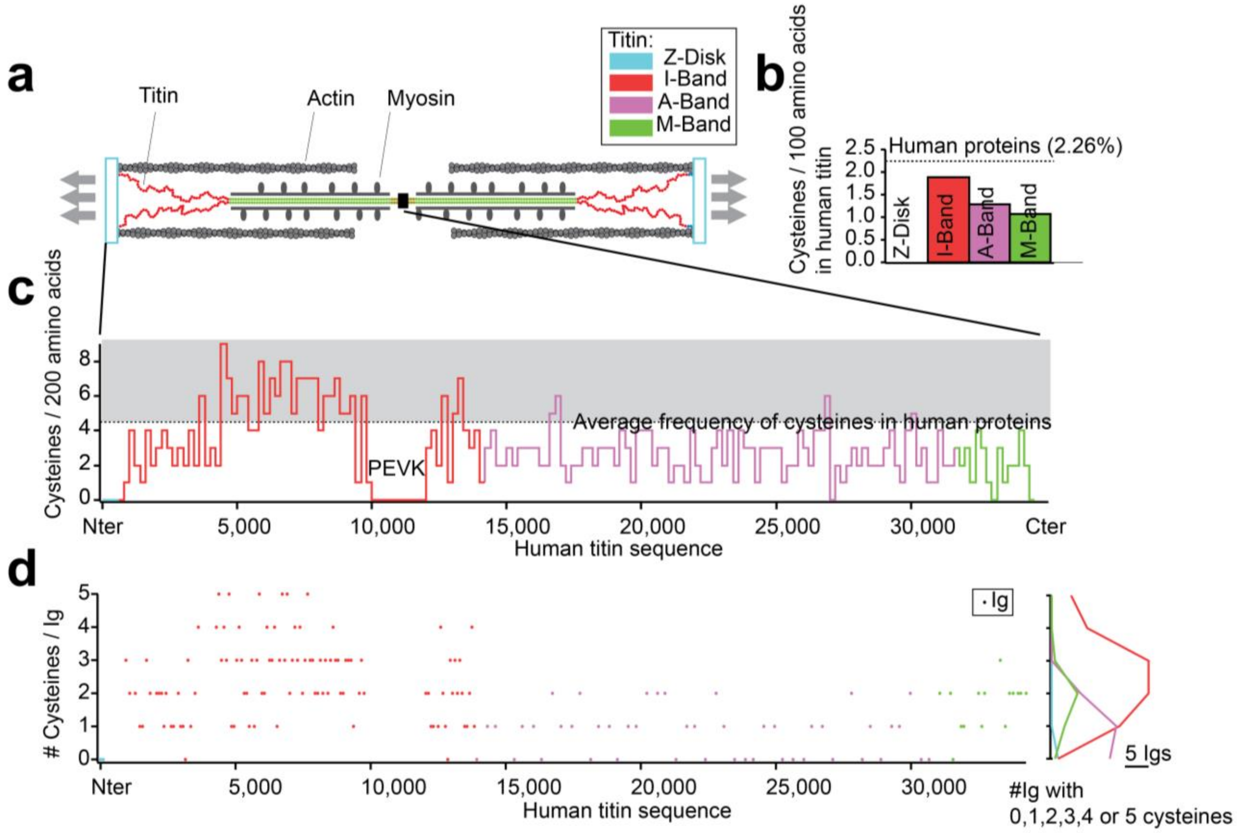

Supplementary Figure 7 – Occurrence of cysteines along the titin sequence and its Ig domains. (a) We use the annotation of domains of the canonical titin sequence (Uniprot: Q8WZ42-1) to define the limits of the different segments of titin along the sarcomere. The Z-disk segment spans from the N-terminus to the residue 740 (last Z-repeat motif). The I-band spans from the residue 741 to 14018 (first fibronectin domain). The A-band spans from the residue 14019 to 32144 (last fibronectin domain). The M-band segment spans from the residue 32144 to C-terminal residue 34350. This nomenclature correlates with the classical description of the sarcomere [20,21]. **(b)** We show in this histogram the percentage of cysteines in the different portions of titin [12,22]. Cysteines are absent in the Z-disk and appear with a frequency per amino acid of 1.90%, 1.30%, 1.09% in the I-, A- and M-band respectively. The average number of cysteines observed in the human proteome is 2.26% [23]. **(c)** Distribution of cysteines along the titin filament. The bin size is 200 amino acids. The I-band shows a peak in the frequency of cysteines that is higher than the average value observed in human protein (grey area). **(d)** Distribution of cysteines along the 163 Ig modules of titin. The histogram highlights that the majority of domains in the I-band have either 2 or 3 cysteines and can contain up to 5 cysteines.

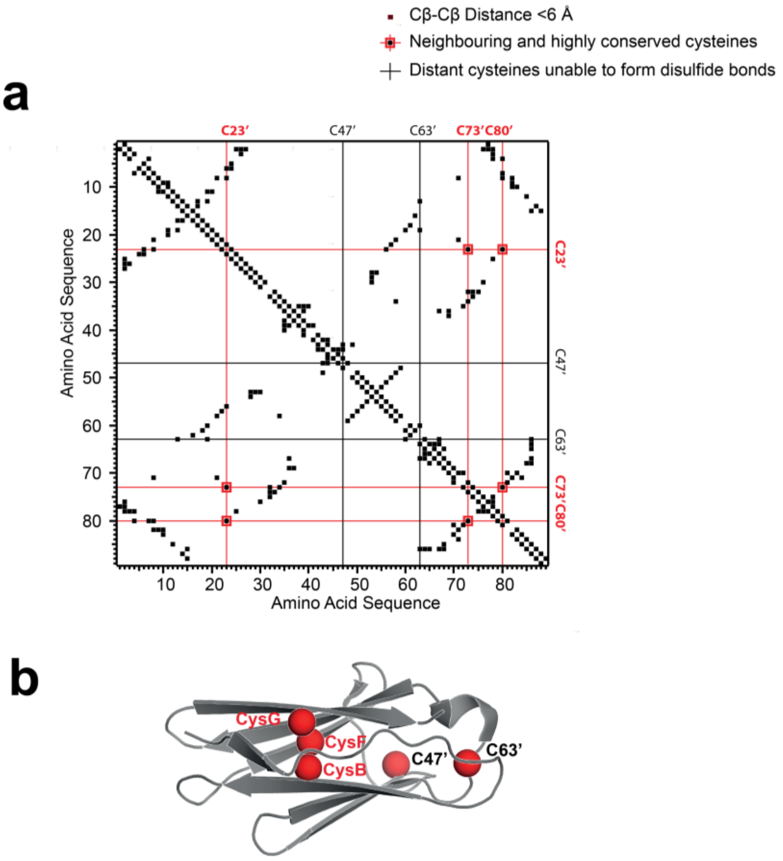

Supplementary Figure 8 – Estimation of cysteine proximity and possible pairing in Ig domains

(a) Prediction of cysteines pairs based on the neighboring position in the structure of the Ig domains. A close Cβ-Cβ distance of two cysteine residues, inferior to 6 Å, suggests that the side chains are close enough to form a cross-link [24]. The X-ray structure of I69 shows that the Cβ-Cβ distance of the linked cysteines is equal to 3.6Å and the Cβ-Cβ distance with the third cysteine C73' is inferior to 6 Å (4.7 Å and 5.5 Å) (Supplementary Figure 1c). The contact map in the figure shows Cβ contacts shorter than 6 Å in the reference structure of I91. The map reveals that the highly conserved cysteines C23' (CysB), C73' (CysF) and C80' (CysG) have the structural ability to form a disulfide bond. **(b)** Mapping of conserved cysteines in the Ig structures. The position of conserved cysteines is mapped on the structure of I91 by a red sphere on Cβ position. The co-localized and most conserved cysteines forming the triad C23'-C73'-C80' are observed in the hydrophobic core and are distant from the other conserved cysteines. The next most conserved cysteines are the distant cysteines C47' and C63' and cannot form a disulfide bond in the native structure [12,14].

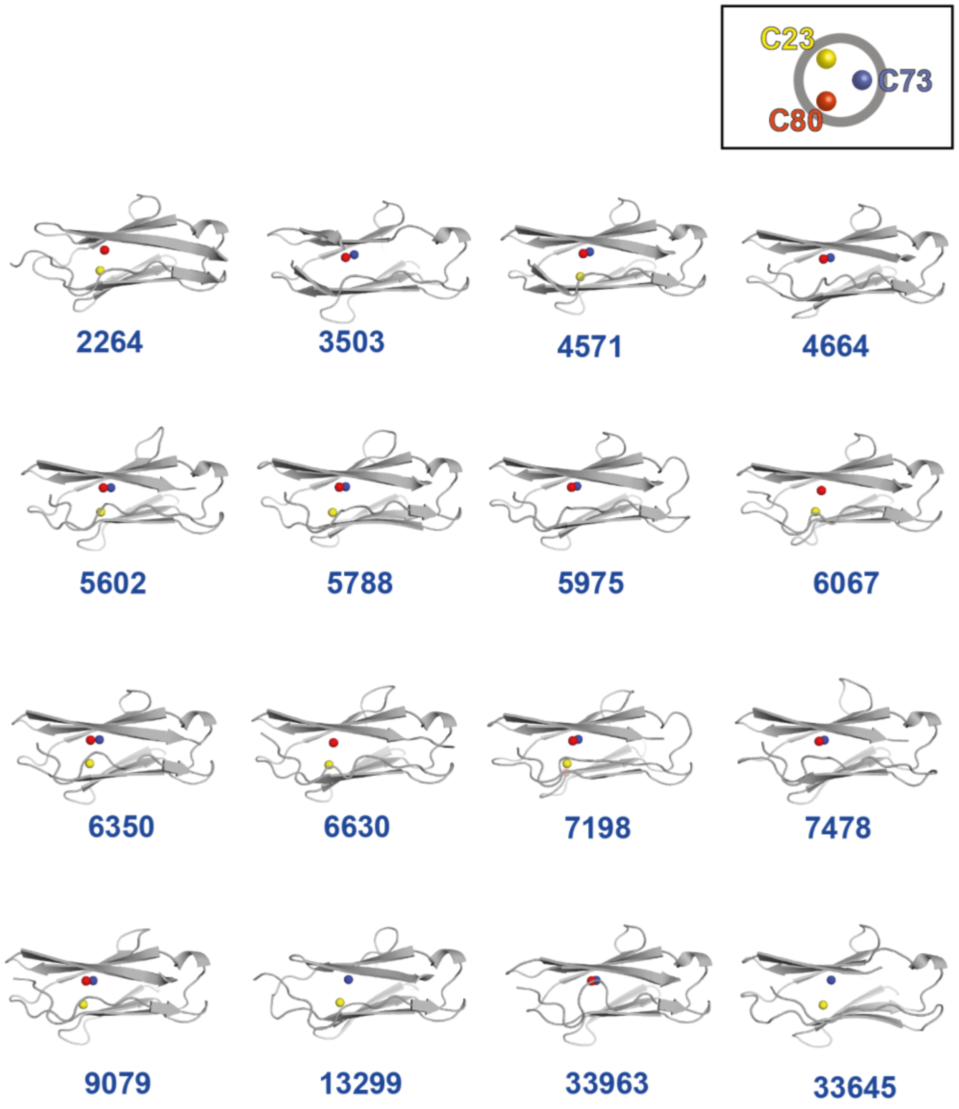

Supplementary Figure 9 – Examples of structures titin domains containing cysteines of the triad

Structural models show different types of cysteine combinations. The last two domains, at positions 33963 and 33645, are located in the M-band whereas the other examples are found in the I-band. Cysteines are represented by colored spheres centered at the Cβ atom. CysB, CysF and CysG are shown respectively in yellow, blue and red. The first amino acid of each domain in the canonical titin sequence (Uniprot: Q8WZ42-1) is indicated.

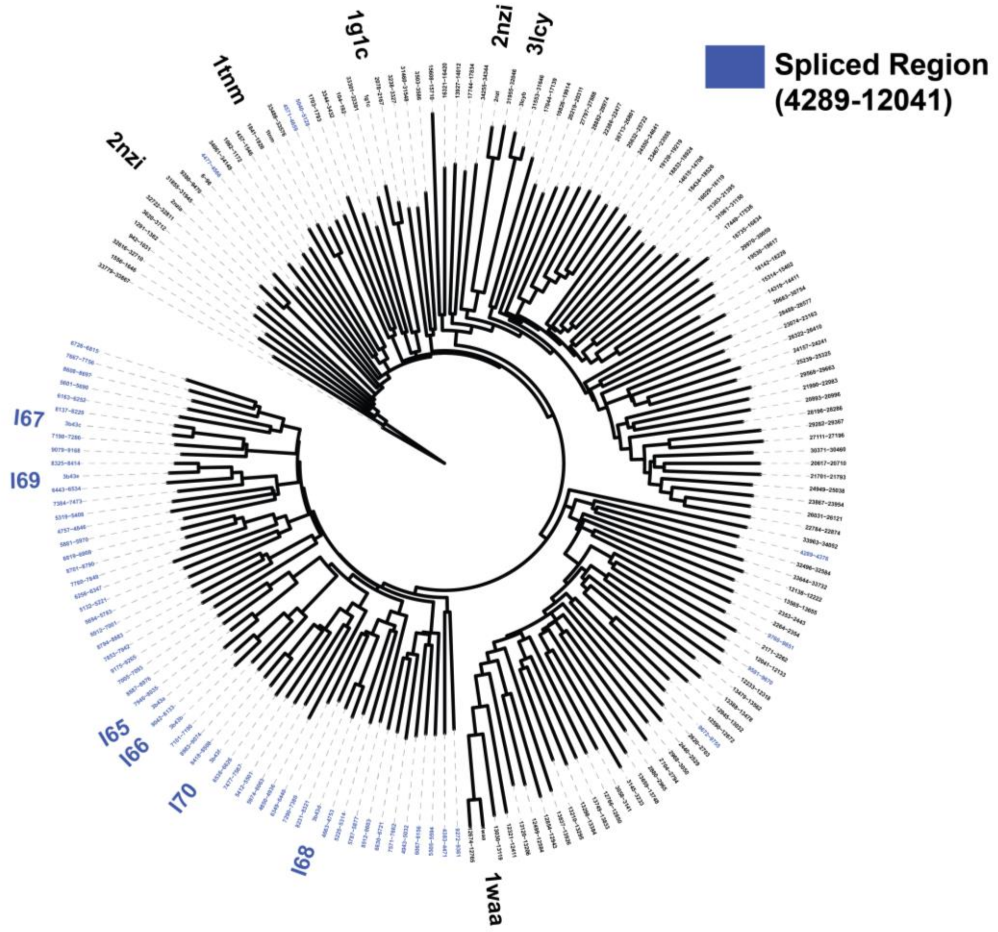

Supplementary Figure 10 – Clustering of titin's Ig domains based on their sequence similarity

We constructed a tree with Itol [25] from the sequence alignment of all titin Ig domains (human canonical sequence of titin, uniprot Q8WZ42) and the 10 sequences used for the structural reconstruction (pdb codes are indicated; the six domains of the I65-I70 fragment in pdb 3B43 are annotated in blue). This representation, comparable to a phylogenetic tree, highlights clusters of similar sequences connected by short branches. For each leaf of the tree, we indicated the boundaries of the associated Ig domain in Q8WZ42. The blue leaves of the tree correspond to Ig domains present in the spliced region located between the amino acid 4289 and 12041. The tree reveals that 51 out of 58 of Ig domains in the differentially spliced region cluster together and share high sequence similarity. The six domains of I65-I70 belong to this cluster and are therefore good representatives of the alternatively spliced domains.

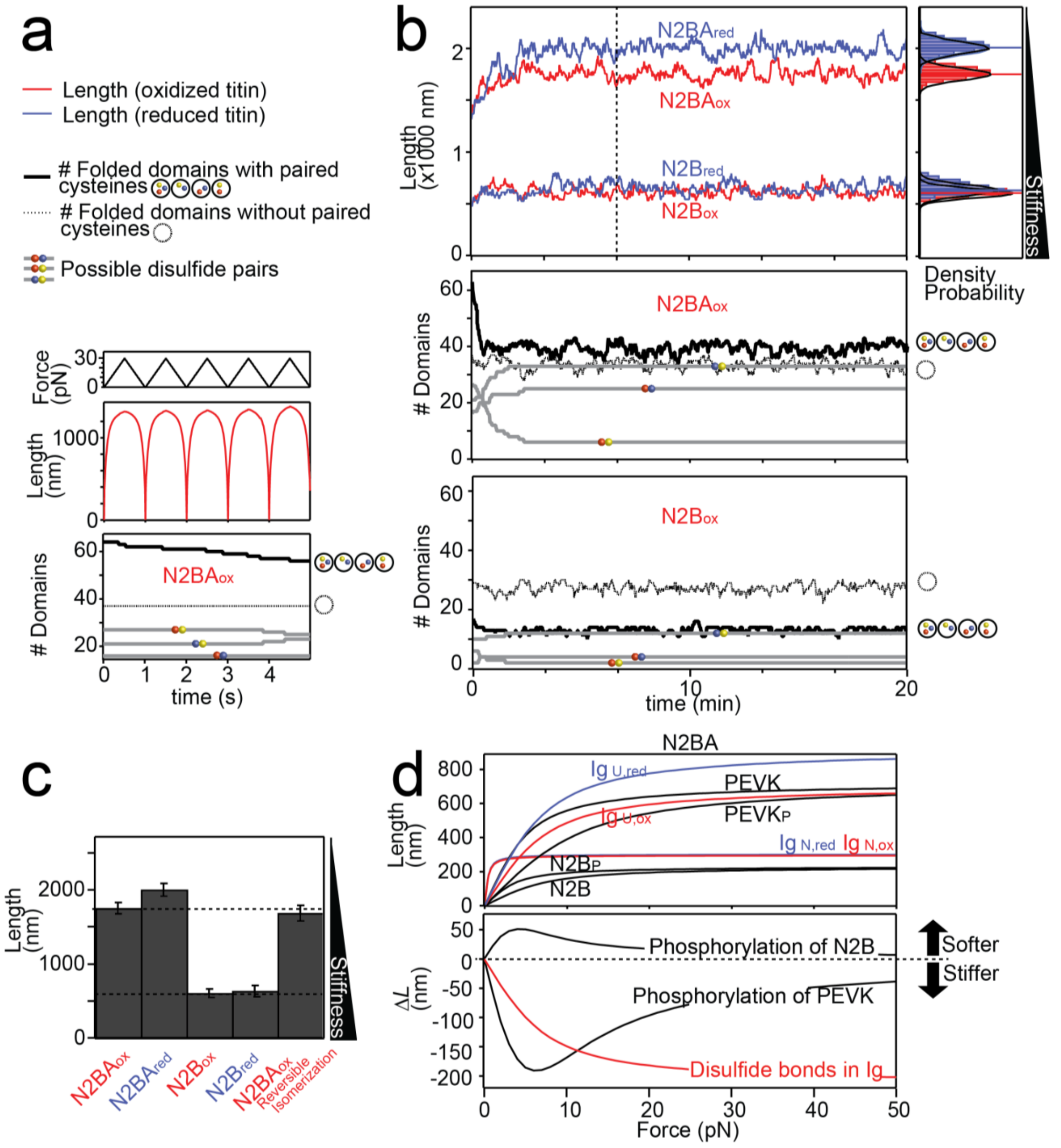

Supplementary Figure 11 – Monte Carlo simulations

(a). First five cycles of a Monte Carlo simulation. The upper graph shows the force protocol applied to the modeled I-band of N2BA titin. The length of the protein (red trace) increases with force as the molecular springs of titin stretch following the freely jointed chain model of polymer elasticity. Recoiling of titin occurs as the force is decreased. Application of force also triggers domain unfolding. This is shown in the bottom panel representing the number of folded domains (black lines), where a total of 8 oxidized domains are captured to unfold (steps down in the solid black line). These unfolding events enable isomerization reactions (grey traces). Note that in these first cycles no unfolding of domains without paired cysteines is detected due to their higher mechanical stability (dotted black line). The change in contour length of titin due to unfolding, folding and isomerization events can be easily followed by monitoring the length of the protein at 30 pN. **(b)** 20-minute Monte Carlo simulations of N2B and N2BA titin isoforms under the force cycle between 0 and 30 pN, both in the reduced and in the oxidized state. The length of the proteins at 30 pN (upper panel) together with the number of folded domains and disulfide populations (lower panels) are shown. On the right, we show length histograms after reaching steady state situation

(from the dotted line, 400 seconds). **(c)** Average protein length for different isoforms and oxidation states. We also tested the effect of disulfide reisomerization, i.e. reformation of the initial CysB-CysG disulfide (last bar in the graph). In this case, we consider that reisomerization occurs at a rate of $0.1s^{-1}$ only when the force is lower than 5pN. **(d)** We used the freely jointed chain model to estimate how different post-translational modifications change the length of N2BA titin at different forces. We considered that phosphorylation of the N2B spring changes the Kuhn length from 1.32 to 2.53 nm [26] and that phosphorylation of the PEVK spring changes the Kuhn length from 1.82 to 0.83 nm [27]. To measure the effect of disulfide bonds on the N2BA contour length, we ran additional simulations and computed the average number of unfolded domains for each oxidation state. These data allowed us to estimate the contour length of folded (Ig_N) and unfolded (Ig_U) Ig domains both in the reduced and oxidized titin. The upper graph shows the resulting force-extension curves calculated using the freely jointed chain for each contributor to elasticity. In the lower panel, we report the differences in length ΔL due post-translational modifications.

Supplementary Tables

Supplementary Table 1- Domain boundaries and cysteine positions in the domains I65-I70 of titin. This table reports the position of the possible disulfide bonds in the 6 domains forming I65-I70 (pdb: 3B43). We indicate the type of cysteines (B, F, or G) involved in the disulfides. *L1* is the number of amino acids that extend upon mechanical unfolding. *L2* corresponds to the number of amino acids that are located between the cysteines forming disulfide bonds [5]. Oxidized cysteines are not included in the calculation of *L1* but are taken into account in the calculation of expected AFM step sizes of unfolding (see Supplementary Note 2). The length of segments unraveling with force in the reduced domains and oxidized domains are indicated respectively in blue and red.

<table>
<tr><th></th><th>SS-Bonds</th><th>first aa</th><th>last aa</th><th>#aa per domain</th><th>L1</th><th>L2</th></tr>
<tr><td rowspan="2">I65</td><td>No SS bond</td><td rowspan="2">3</td><td rowspan="2">90</td><td rowspan="2">88</td><td>88</td><td>0</td></tr>
<tr><td>23-74 (CysB-CysF)</td><td>36</td><td>51</td></tr>
<tr><td rowspan="2">I66</td><td>No SS bond</td><td rowspan="2">99</td><td rowspan="2">188</td><td rowspan="2">90</td><td>90</td><td>0</td></tr>
<tr><td>119-170 (CysB-CysF)</td><td>38</td><td>51</td></tr>
<tr><td rowspan="4">I67</td><td>No SS bond</td><td rowspan="4">195</td><td rowspan="4">282</td><td rowspan="4">88</td><td>88</td><td>0</td></tr>
<tr><td>215-266 (CysB-CysF)</td><td>36</td><td>51</td></tr>
<tr><td>215-277 (CysB-CysG)</td><td>25</td><td>62</td></tr>
<tr><td>266-277 (CysF-CysG)</td><td>76</td><td>11</td></tr>
<tr><td rowspan="2">I68</td><td>No SS bond</td><td rowspan="2">288</td><td rowspan="2">375</td><td rowspan="2">88</td><td>88</td><td>0</td></tr>
<tr><td>309-360 (CysB-CysF)</td><td>36</td><td>51</td></tr>
<tr><td rowspan="4">I69</td><td>No SS bond</td><td rowspan="4">382</td><td rowspan="4">469</td><td rowspan="4">88</td><td>88</td><td>0</td></tr>
<tr><td>402-453 (CysB-CysF)</td><td>36</td><td>51</td></tr>
<tr><td>402-464 (CysB-CysG)</td><td>25</td><td>62</td></tr>
<tr><td>453-464 (CysF-CysG)</td><td>76</td><td>11</td></tr>
<tr><td rowspan="2">I70</td><td>No SS bond</td><td rowspan="2">475</td><td rowspan="2">562</td><td rowspan="2">88</td><td>88</td><td>0</td></tr>
<tr><td>547-558 (CysF-CysG)</td><td>76</td><td>11</td></tr>
</table>

Supplementary Table 2 - Calculation of steps due to unfolding. Steps were calculated according to:

$\Delta x_{unfolding}$ (force) = $W_{wlc\ (force)}$ *[$L1$ * 0.4 – 3.0 + 0.8*nSS]

$\Delta x_{unfolding}$ (nm)	100pN	170pN
No SSbond	26.1	27.4
CysB-CysG	6.3	6.6
CysB-CysF	9.9	10.4
CysF-CysG	22.8	24.0

Supplementary Table 3 - Calculation of steps due to disulfide isomerization. Steps were calculated according to:

$\Delta x_{isomerization}$ (force) = $W_{wlc\ (force)}$ * $\Delta L1$* 0.4

$\Delta x_{isomerization}$ (nm)	100pN	170pN
CysB-CysF (ΔL1=11)	3.6	3.7
CysF-CysG (ΔL1=51)	16.5	17.3

Suppementary Table 4 - Calculation of steps due to disulfide reduction. Steps were calculated according to:

$\Delta x_{reduction}$ (force) = $W_{wlc\ (force)}$ * [$L2$* 0.4 - 0.8]

$\Delta x_{reduction}$ (nm)	100pN	170pN
CysB-CysG	19.4	20.4
CysB-CysF	15.9	16.7
CysF-CysG	2.9	3.1

Suppementary Table 5 – Distribution of CysB, CysF and CysG in the I-band of titin. The last column indicates the number of amino acids of the isoform. The theoretical non-spliced titin contains 38,138 residues. See also Supplementary Note 6.

Isoform	# of Igs with no paired CysB, CysF or CysG	# of Igs with CysB-CysF	# of Igs with CysF-CysG	# of Igs with CysB-CysG	# of Igs with triad (percentage of total)	Any disulfide involving CysB, CysF or CysG, in %	#amino acids
N2BA ***(Q8WZ42-1)***	37	21	16	6	21 (21%)	63%	34350
N2B ***(Q8WZ42-3)***	30	10	2	2	4 (8%)	37%	26926
Soleus ***(Q8WZ42-4)***	36	21	14	6	21 (21%)	63%	33445
Novex-3 ***(Q8WZ42-6)***	20	4	5	2	6 (16%)	46%	5604
Novex-2 ***(Q8WZ42-7)***	36	21	15	6	21 (21%)	64%	33615

Supplementary Table 6 –Mechanical parameters for Monte Carlo simulations

Contour lengths and Kuhn lengths of the different regions of titin have been reported before [11,12]. The contour length of disulfide-containing unfolded domains where calculated considering a 0.4 nm contour length per amino acid and 0.8 nm contour length per disulfide bond [3,5]. The following table summarizes the values of contour lengths and Kuhn lengths used in the simulations:

<table>
<tr><th></th><th></th><th>Contour Length (nm)</th><th>Kuhn Length (nm)</th></tr>
<tr><td rowspan="5">Parameters Ig</td><td>folded (N)</td><td>4</td><td>20</td></tr>
<tr><td>Ured</td><td>35.2</td><td>1.32</td></tr>
<tr><td>UB-G</td><td>10.8</td><td>1.32</td></tr>
<tr><td>UB-F</td><td>15.2</td><td>1.32</td></tr>
<tr><td>UF-G</td><td>31.2</td><td>1.32</td></tr>
<tr><td rowspan="2">Parameters Elastic Spring Regions</td><td>N2B region</td><td>230</td><td>1.32</td></tr>
<tr><td>PEVK region</td><td>N2BA isoform: 721
N2B isoform: 68</td><td>1.82</td></tr>
</table>

Supplementary Table 7–Transition rates for Monte Carlo simulations

In the table below, we report the parameters used for the force-dependent transition rates between folded (N) and unfolded (U) states. Disulfide bonding is indicated by the corresponding cysteine positions B, F and G. Parameters are obtained from the literature [9] or estimated according to our results. Simulations assume that the force dependency of the reactions is described by the Bell model [13]. We consider that the unfolding rate of oxidized domains is the same independently of the specific disulfide present in the domain. We estimated folding rates of domains containing disulfides B-G and B-F from data in Figure 5D considering that the folding reaction follows zero-order kinetics. To estimate the folding rate of reduced domains containing paired cysteines, we considered a 28x reported acceleration of folding induced by disulfide bonds [14]. Isomerization reactions were considered to be force dependent with the same Δx as for the reduction of a disulfide bond by L-Cys [15]. We also ran simulations in which we enable isomerization reactions to UB-G from UB-F and UF-G at forces below 5 pN and at a rate of 0.1 s^{-1}.

	transitions		Parameters	
Type of Igs	Initial state	Final state	Δx (nm)	α_0 (s^{-1})
Non paired cysteines (I91 – like)	Nred	Ured	0.25	8.00E-05
	Ured	Nred	-2.2	1.2
Paired cysteines (B, F, G)	un/folding with reduced cysteines			
	Nred	Ured	0.12	4.40E-03
	Ured	Nred	-2.2	2.04E-3
	un/folding with disulfide bonds			
	NB-G	UB-G	0.12	4.66E-02
	NB-F	UB-F	0.12	4.66E-02
	NF-G	UF-G	0.12	4.66E-02
	UB-G	NB-G	-2.2	0.057
	UB-F	NB-F	-2.2	0.0544
	UF-G	NF-G	-2.2	2.04E-3
	Isomerization			
	UB-G	UB-F	0.023	0.012
	UB-G	UF-G	0.023	0.015

Supplementary Notes

Supplementary Note 1 – Sequence alignment of rabbit and human I65-I70 fragments of titin

The rabbit sequence corresponds to the pdb structure 3B43. The human sequence is the fragment 7945-8511 in uniprot Q8WZ42-1. This alignment shows 95% sequence identity between the I65-I70 fragments, the absence of inserts, and the strict conservation of all 14 cysteines.

```
Rabbit          ----EPPYFIEPLEHVEAAIGEPITLQCKVDGTPEIRIAWYKEHTKLRSAPAYKMQFKNNVASLVINKVDHSDVGEYTCK
human           ----EPPYFIEPLEHVEAVIGEPATLQCKVDGTPEIRISWYKEHTKLRSAPAYKMQFKNNVASLVINKVDHSDVGEYSCK
                    **************.**** **************:**************************************:**

Rabbit          AENSVGAVASSAVLVIKERKLPPSFARKLKDVHETLGFPVAFECRINGSEPLQVSWYKDGELLKDDANLQTSFIHNVATL
human           ADNSVGAVASSAVLVIKARKLPPFFARKLKDVHETLGFPVAFECRINGSEPLQVSWYKDGVLLKDDANLQTSFVHNVATL
                *:*************** ***** ************************************ ************:******

Rabbit          QILQTDQSHVGQYNCSASNPLGTASSSAKLTLSEHEVPPFFDLKPVSVDLALGESGTFKCHVTGTAPIKITWAKDNREIR
human           QILQTDQSHIGQYNCSASNPLGTASSSAKLILSEHEVPPFFDLKPVSVDLALGESGTFKCHVTGTAPIKITWAKDNREIR
                *********:******************** *************************************************

Rabbit          PGGNYKMTLVENTATLTVLKVTKGDAGQYTCYASNVAGKDSCSAQLGVQEPPRFIKKLEPSRIVKQDEHTRYECKIGGSP
human           PGGNYKMTLVENTATLTVLKVGKGDAGQYTCYASNIAGKDSCSAQLGVQEPPRFIKKLEPSRIVKQDEFTRYECKIGGSP
                ********************* *************:********************************.***********

Rabbit          EIKVLWYKDETEIQESSKFRMSFVESVAVLEMYNLSVEDSGDYTCEAHNAAGSASSSTSLKVKEPPVFRKKPHPVETLKG
human           EIKVLWYKDETEIQESSKFRMSFVDSVAVLEMHNLSVEDSGDYTCEAHNAAGSASSSTSLKVKEPPIFRKKPHPIETLKG
                ************************:*******:*********************************:*******:*****

Rabbit          ADVHLECELQGTPPFQVSWHKDKRELRSGKKYKIMSENFLTSIHILNVDSADIGEYQCKASNDVGSDTCVGSITLKAPPR
human           ADVHLECELQGTPPFHVSWYKDKRELRSGKKYKIMSENFLTSIHILNVDAADIGEYQCKATNDVGSDTCVGSIALKAPPR
                ***************:***:*****************************:**********:************:******

Rabbit          FVKKLSDISTVVGEEVQLQATIEGAEPISVAWFKDKGEIVRESDNIWISYSENIATLQFSRAEPANAGKYTCQIKNEAGT
human           FVKKLSDISTVVGKEVQLQTTIEGAEPISVVWFKDKGEIVRESDNIWISYSENIATLQFSRVEPANAGKYTCQIKNDAGM
                *************:*****:**********.******************************.**************:** 

Rabbit          QECFATLSVLE
human           QECFATLSVLE
                ***********
```

Supplementary Note 2 – Calculation of expected extensions.
We seek to calculate all the theoretical extensions addressed in this study. First, we estimate the number of amino acids that extend upon mechanical unfolding by analyzing the structure of the 6 different domains included in the I65-I70 fragment (pdb: 3B43). Supplementary Table 1 shows that the six domains are highly similar in terms of structure and sequence length. For the characterization of the domain boundaries and sequence length (#aa per domain), we consider the first and last hydrogen bonds that participate in the mechanical clamp motif formed between the β-strands A and G [1,2]. All domains have 88 amino acids protected by the mechanical clamp, except I66 (90 amino acids).

In oxidized domains, disulfide bonds cannot be cleaved by force and limit the extension of the polypeptide since the polypeptide chain located between the linked cysteines does not experience mechanical force [3]. Supplementary Table 1 displays that the amino acid length of the segments delimited by the cysteines are equal in the six domains. In conclusion, the unfolding of I65-I70 domains should give rise to consistent steps, both in the absence of disulfide bond [4] or in the case of equivalent disulfide pairs.

In our experiments, we stretch the polyproteins applying a constant force. We use the Worm-Like Chain model of polymer elasticity to calculate the actual extensions at a given force from the corresponding number of amino acids that extend (Supplementary Table 1) [6]. At 100pN and 170pN, the extension of the polyprotein corresponds respectively to 81% and 85% (W_{wlc}) of the contour length considering a typical persistence length of 0.3 nm [7,8]. We used the equations presented in Supplementary Tables 2-4 to calculate the theoretical step sizes $\Delta x_{\text{unfolding}}$, $\Delta x_{\text{isomerization}}$, and $\Delta x_{\text{reduction}}$ that result from the different events [5]. In the equations in Supplementary Tables 1-4, *L1* is the number of amino acids mechanically protected by the mechanical clamp, 3 nm is the end-to-end contour length of a folded domain, and *nSS* is the number of disulfide bonds. The contour length per amino acid (0.4 nm) [3] and of a cystine crosslink (0.8 nm) [5] have been estimated before. For simplicity, in the calculations we assume that domain I66 also has 88 amino acids protected by the clamp (Supplementary Table 1).

Supplementary Note 3 – Multiple Alignment of Ig domains

```
                                       C23'(CysB)                                                                      C73'(CysF)   Cys80'(CysG)
0006 -----PTFTQPLQS-----VVVLEGS----TATFEAHIS--GFPVPEVSWFRD-G--------QVIST-STL PGVQISF-SDGRAKLTIPAVTKA-NSGRYSLKATNGS-GQATSTAELL------
0104 ----PPNFVQRLQS-----MTVRQGS----QVRLQVRVT--GIPTPVVKFYRD-G--------AEIQS-S-- LDFQISQ-EGDLYSLLIAEAYPE-DSGTYSVNATNSV-GRATSTAELL------
0943 ----PPTLVSGLKN-----VTVIEGE----SVTLECHIS--GYPSPTVTWYRE-D--------YQIES-SID FQITFQS---GIARLMIREAFAE-DSGRFTCSAVNEA-GTVSTSCYLA------
1082 ----PPYFITKPVV-----QKLVEGG----SVVFGCQVG--GNPKPHVYWSKS-G--------VPLTT-GYR YKVSYNK-QTGECKLVISMTFAD-DAGEYTIVVRNKH-GETSASASLL------
1291 -----SGFDSRIKN-----YRILEGM----GVTFHCKMS--GYPLPKIAWYKD-G--------KRIKH-GER YQMDFLQ--DGRASLRIPVVLPE-DEGIYTAFASNIK-GNAICSGKLYVE----
1457 ----PVFVLKPVS-----FKCLEGQ----TARFDLKVV--GRPMPETFWFHD-G--------QQIVN-DYT HKVVI-K-EDGTQSLIIVPATPS-DSGEWTVVAQNRA-GRSSISVILT------
1556 ----PMFVEKLKN-----VNIKEGS----RLEMKVRAT--GNPNPDIVWLKN-S--------DIIVP-HKY PKIRIEG-TKGEAALKIDSTVSQ-DSAWYTATAINKA-GRDTTRCKVN------
1703 -----PFFKKKLTS-----LRLKRFG----PAHFECRLTPIGDPTMVVEWLHD-G--------KPLEA-AN- RLRMINE-FG-YCSLDYGVAYSR-DSGIITCRATNKY-GTDHTSATLI------
1841 ----PDIVLYPEP-----VRVLEGE----TARFRCRVT--GYPQPKVNWYLN-G--------QLIRK-SKR FRVRY---DGIH-YLDIVDCKSY-DTGEVKVTAENPE-GVIEHKVKLE------
2078 ----PKIFERIQS-----QTVGQGS----DAHFRVRVV--GKPDPECEWYKN-G--------VKIER-SD- RIYWYWP-EDNVCELVIRDVTAE-DSASIMVKAINIA-GETSSHAFLL------
2171 --KQLITFTQELQD-----VVAKEKD---TMATFECETSEPFVK-VK--WYKDG---------MEVH---EG DKYRMHS-DRKVHFLSILTIDTS-DAEDYSCVLVEDEN--VKTTAKLIV-----
2264 --GAVVEFVKELQD-----IEVPESY----SGELECIVS---PENIEGKWYHN---------DVELKS---N GKYTITS-RRGRQNLTVKDVTKE-DQGEYSFVIDGKK---TTCKLKMKPR----
2353 --PRPIAILQGLSD-----QKVCEGD----IVQLEVKVS---LESVEGVWMKDGQ---------EVQP---S DRVHIVI-DKQSHMLLIEDMTKE-DAGNYSFTIPALG-LSTSGRVSVY------
2430 VSVYSVDVITPLKD-----VNVIEGT----KAVLECKVSVPDVTSVK--WYLND---------EQIK---PD DRVQAIV-KGTKQRLVINRTHAS-DEGPYKLIVG-----RVETNCNLS------
2620 -----GAISKPLTD-----QTVAESQ----EAVFECEVANPDSK-GE--WLRDG---------KHLP---LT NNIRSES-DGHKRRLIIAATKLD-DIGEYTYKVA-----TSKTSAKLK------
2704 --VEAVKIKKTLKN-----LTVTETQ----DAVFTVELTHPNVKGVQ--WIKNG---------VVLE---SN EKYAISV-KGTIYSLRIKNCAIV-DESVYGFRLG-----RLGASARLHVE----
2880 --ETLHITKTMKN-----IEVPETK----TASFECEVSHFNVP-SM--WLKNG---------VEIE---MS EKFKIVV-QGKLHQLIIMNTSTE-DSAEYTFVCG-----NDQVSATLT------
2968 ----PIMITSMLKD-----INAEEKD----TITFEVTVNYEGIS-YK--WLKNG---------VEIK---ST DKCQMRT-KKLTHSLNIRNVHFG-DAADYTFVAG-----KATSTAT--------
3058 -----IEFRKHIKD-----IKVLEKK----RAMFECEVSEPDIT-VQ--WMKD----------DQELQ---IT DRIKIQK-EKYVHRLLIPSTRMS-DAGKYTVVAG-----GNVSTAKLF------
3145 -EGRDVRIRSIKKE-----VQVIEKQ----RAVVEFEVNEDDVD-AH--WYKDGI--------EINFQ---VQ ERHKYVV-ERRIHRMFISETRQS-DAGEYTFVAG-----RNRSSVTLYV-----
3239 ----PPQVLQELQP-----VTVQSGK----PARFCAVIS--GRPQPKISWYKE-E--------QLLST-GFK CKFLHDG--QE-YTLLLIEAFPE-DAAVYTCEAKNDY-GVATTSASLS------
3344 ----PPAIITPLQD-----TVTSEGQ----PARFQCRVS--GTDLKVSWYSKD---------KKIKP-S-- RFFRMTQ-FEDTYQLEIAEVYPE-DEGTYTFVASNAV-GQVSSTANLS------
3503 -----PIFIKEVSN-----ADISMGD----VATLSVTVI--GIPKPKIQWFFN-G--------VLLTP-SAD YKFVFDG--DD-HSLIILFTKLE-DEGEYTCMASNDY-GKTIC----------
3621 ----PPHFLKELKP-----IRCAQGL----PAIFEYTVV--GEPAPTVTWFKE-N--------KQLCT-SV- YYTIIHN-PNGSGTFIVNDPQRE-DSGLYICKAENML-GESTCAAELLVL----
4289 ----PMIHTPLVD-----TVSEEGD----IVHLTTSIT----NAKEVNWYFENK---------LVPS---S EKFKCLQ-DQNTYTLVIDKVNTEDHQGEYVCEALNDS-GKTATSAKLT------
4383 ----PVIKRKIEP-----LEVALGH----LAKFTCEIQ--SAPNVRFQWFKA-G--------REIYE---S DKCSIRS-SKYISSLEILRTQVV-DCGEYTCKASNEY-GSVSCTATLT------
4478 ----PPTFLSRPKS-----LTTFVGK----AAKFICTVT--GTPVIETIWQKD-G--------AALSP---S PNWKISD-AENKHILELSNLTIQ-DRGVYSCKASNKF-GADICQAELI------
4571 ----PHFIKELEP-----VQSAINK----KVHLECQVD--EDRKVTVTWSKD-G--------QKLPP-GK- DYKICFE-DK-IATLEIPLAKLK-DSGTYVCTASNEA-GSSSCSATVT------
4664 ----PPSFVKKVDPS----YLMLPGE----SARLHCKLK--GSPVIQVTWFKN-N--------KELSE---S NTVRMYF-VNSEAILDITDVKVE-DSGSYSCEAVNDV-GSDSCSTEIV------
4758 ----PPSFIKTLEP-----ADIVRGT----NALLQCEVS--GTGPFEISWFKD-K--------KQIRS---S KKYRLFS-QKSLVCLEIFSFNSA-DVGEYECVVANEV-GKCGCMATHL------
4851 ----PPTFVKKVDDL----IALG-GQ----TVTLQAAVR--GSEPISVTWMKG-Q--------EVIRE---S GKIKMSF-SNGVAVLIIPDVQIS-FGGKYTCLAENEA-GSQTSVG---------
4943 ----PAKIIERAEL-----IQVTAGD----PATLEYTVA--GTPELKPKWYKD-G--------RPLVA---S KKYRISF-KNNVAQLKFYSAELH-DSGQYTFEISNEV-GSSSCETTFT------
5040 -----PFFTKPLRN-----VDSVVNG----TCRLDCKIA--GSLPMRVSWFKD-G--------KEIAA-SD- RYRIAFV-EG-TASLEIIRVDMN-DAGNFTCRATNSV-GSKDSSGALI------
5133 ----PPSFVTKPGS-----KDVLPGS----AVCLKSTFQ--GSTPLTIRWFKG-N--------KELVS---G GSCYITK-EALESSLELYLVKTS-DSGTYTCKVSNVA-GGVECSANLF------
5225 ----PATFVEKLEPS----QLLKKGD----ATQLACKVT--GTPPIKITWFAN-D--------REIKE---S SKHRMSF-VESTAVLRLTDVGIE-DSGEYMCEAQNEA-GSDHCSSIV-------
5320 ----PPYFTKEFKP-----IEVLKEY----DVMLLAEVA--GTPPFEITWFKD-N--------TILRS---G RKYKTFI-QDHLVSLQILKFVAA-DAGEYQCRVTNEV-GSSICSARVT------
5413 ----PPSFIKKIEST----SSLR-GG----TAAFQATLK--GSLPITVTWLKD-S--------DEITE---S DNIRMTF-ENNVASLYLGKVE-HDGKYVCQAKNDA-GIQRCSALLS------
5505 ----PATITEEAVS-----IDVTQGD----PATLQVKFS--GTKEITAKWFKD-G--------QELTL---G SKYKISV-TDTVSILKIISTEKK-DSGEYTFEVQNDV-GRSSCKARIN------
5602 ----PPSFTKKLKK-----MDSIKGS----FIDLECIVA--GSHPISIQWFKD-D--------QEISA---S EKYKFSF-HDNTAFLEISQLEGT-DSGTYTCSATNKA-GHNQCSGHLT------
5695 ----PPYFVEKPQS-----QDVNPNT----RVQLKALVG--GTAPMTIKWFKD-N--------KELHS---G AARSVWK-DDTSTSLELFAAKAT-DSGTYICQLSNDV-GTATSKATLF------
5788 ----PPQFIKKPSPV----LVLRNGQ----STTFECQIT--GTPKIRVSWYLD-G--------NEITA---I QKHGISF-IDGLATFQISGARVE-NSGTYVCEARNDA-GTASCSIELK------
5882 ----PPTFIRELKP-----VEVVKYS----DVELECEVT--GTPPFEVTWLKN-N--------REIRS---S KKYTLTD-RVSVFNLHITKCDPS-DTGEYQCIVSNEG-GSCSCSTRVA------
5975 ----PPSFIKKIENT----TTVL-KS----SATFQSTVA--GSPPISITWLKD-D--------QILDE---D DNVYISF-VDSVATLQIRSVDNG-HSGRYTCQAKNES-GVERCYAFLL------
6067 ----PAQIVEKAKS-----VDVTEKD----PMTLECVVA--GTPELKVKWLKD-G--------KQIVP---S RYFSMSF-ENNVASFRIQSVMKQ-DSGQYTFKVENDF-GSSSCDAYLR------
6164 ----PPSFTKKLTK-----MDKVLGS----SIHMECKVS--GSLPISAQWFKD-G--------KEIST---S AKYRLVC-HERSVSLEVNNLELE-DTANYTCKVSNVA-GDDACSGILT------
6257 ----PPSFLVKPGR-----QQAIPDS----TVEFKAILK--GTPPFKIKWFKD-D--------VELVS---G PKCFIGL-EGSTSFLNLYSVDAS-KTGQYTCHVTNDV-GSDSCTTMLLVT----
6350 ----PPKFVKKLEAS----KIVKAGD----SSRLECKIA--GSPEIRVVWFRN-E--------HELPA---S DKYRMTF-IDSVAVIQMNNLSTE-DSGDFICEAQNPA-GSTSCSTKVIV-----
6440 ----PPVFSSFPPI-----VETLKNA----EVSLECELS--GTPPFEVVWYKD-K--------RQLRS---S KKYKIAS-KNFHTSIHILNVDTS-DIGEYHCKAQNEV-GSDTCVCTVKLK----
6537 ----PPRFVSKLNSL----TVVA-GE----PAELQASIE--GAQPIFVQWLKEKE--------EVIRE---S ENIRITF-VENVATLQFKAAEPA-NAGKYICQIKNDG-GMEENMATLM------
6630 ----PAVIVEKAGP-----MTVTVGE----TCTLECKVA--GTPELSVEWYKD-G--------KLLTS---S QKHKFSF-YNKISSLRILSVERQ-DAGTYTFQVQNNV-GKSSCTAVVDVS----
6727 ----PPSFTRRLKN-----TGGVLGA----SCILECKVA--GSSPISVAWFKE-K--------TKIVS---G AKYQTTF-SDNVCTLQLNSLDSS-DMGNYTCVAANVA-GSDECRAVLT------
6820 ----PPSFVKEPEP-----LEVLPGK----NVTFTSVIR--GTPPFKVNWFRG-A--------RELVK---G DRCNIYF-EDTVAELELFNIDIS-QSGEYTCVVSNNA-GQASCTTRLF------
6912 ----PAAFLKRLSD-----HSVEPGK----SIILESTYT--GTLPISVTWKKD-G--------FNITT---S EKCNIVT-TEKTCILEILNSTKR-DAGQYSCEIENEA-GRDVCGALVS------
7005 ----PPYFVTELEPL----EAAV-GD----SVSLQCQVA--GTPEITVSWYKG-D--------TKLRP---T PEYRTYF-TNNVATLVFKLVNIN-DSGEYTCKAENSI-GTASSKTVF-------
7102 ----PPSFARQLKDI----EQTV-GL----PVTLTCRLN--GSAPIQVCWYRD-G--------VLLRD---D ENLQTSF-VDNVATLKLQTDLS-HSGQYSCSASNPL-GTASSSARLT------
7198 ----PFFDIKPVS-----IDVIAGE----SADFECHVT--GAQPMRITWSKD-N--------KEIRP---G GNYTITC-VGNTPHLRILKVGKG-DSGQYTCQATNDV-GKDMCSAQLS------
7291 ----PPKFVKKLEAS----KVAKQGE----SIQLECKIS--GSPEIKVSWFRN-D--------SELHE---S WKYNMSF-INSVALLTINEASAE-DSGDYICEAHNGV-GDASCSTALT------
7385 ----PPVFTQKPSP-----VGALKGS----DVILQCEIS--GTPPFEVVWVKD-R--------KQVRN---S KKFKITS-KHFDTSLHILNLEAS-DVGEYHCKATNEV-GSDTCSCSVK------
7478 ----PPRFVKKLSDT----STLI-GD----AVELRAIVE--GFQPISVVWLKDRG--------EVIRE---S ENTRISF-IDNIATLQLGSPEAS-NSGKYICQIKNDA-GMRECSAVLT------
7571 ----PARIIEKPEP-----MTVTGN-----PFALECVVT--GTPELSAKWFKD-G--------RELSA---S SKHHITF-INKVASLKIPCAEMS-DKGLYSFEVKNSV-GKSNCTVSVHVS----
7668 ----PPSFIRKLKD-----VNAILGA----SVVLECRVS--GSAPISVGWFQD-G--------NEIVS---G PKCQSSF-SENVCTLNLSLLEPS-DTGIYTCVAANVA-GSDECSAVLT------
7761 ----PPSFEQTPDS-----VEVLPGM----SLTFTSVIR--GTPPFKVKWFKG-S--------RELVP---G ESCNISL-EDFVTELELFEVQPL-ESGDYSCLVTNDA-GSASCTTHLF------
7853 ----PATFVKRLAD-----FSVETGS----PIVLEATYT--GTPPISVSWIKD-E--------YLISQ---S ERCSITM-TEKSTILEILESTKR-DYAQYSCLIENEA-GQDICEALVS------
7946 ----PPYFIEPLEHV----EAVI-GE----PATLQCKVD--GTPEIRISWYKE-H--------TKLRS---A PAYKMQF-KNNVASLVINKVDHS-DVGEYSCKADNSV-GAVASSAVLV------
8042 ----PPFFARKLKDV----HETL-GF----PVAFECRIN--GSEPLQVSWYKD-G--------VLLKD---D ANLQTSF-VHNVATLQILQTDQS-HIGQYNCSASNPL-GTASSSAKLILS----
8138 ----PPFFDLKPVS-----VDLALGE----SGTFKCHVT--GTAPIKITWAKD-N--------REIRP---G GNYKMTL-VENTATLTVKGVKG-DAGQYTCYASNIA-GKDSCSAHLGVQ----
8232 ----PPRFIKKLEPS----RIVKQDE----FTRYECKIG--GSPEIKVLWYKD-E--------TEIQE---S SKFRMSF-VDSVAVLEMHNLSVE-DSGDYTCEAHNAA-GSASSSTSLK------
8326 ----PPIFRKKPHP-----IETLKGA----DVHLECELQ--GTPPFHVSWYKD-K--------RELRS---G KKYKIMS-ENFLTSIHILNVDAA-DIGEYQCKATNDV-GSDTCVGSIA------
8419 ----PPRFVKKLSDI----STVV-GK----EVQLQTTIE--GAEPISVVWFKDKG--------EVIRE---S DNIWISY-SENIATLQFSRVEPA-NAGKYTCQIKNDA-GMQECFATLS------
8512 ----PATIVEKPES-----IKVTTGD----TCTLECTVA--GTPELSTKWFKD-G--------KELTS---D NKYKISF-FNKVSGLKIINVAPS-DSGVYSFEVQNPV-GKDSCTASLQVS----
8609 ----PPSFTRKLKE-----TNGLSGS----SVVMECKVY--GSPPISVSWFHE-G--------NEISS---G RKYQTTL-TDNTCALTVNMLEES-DSGDYTCIATNMA-GSDECSAPLT------
8702 ----PPSFVQKPDP-----MDVLTGT----NVTFTSIVK--GTPPFSVSWFKG-S--------SELVP---S DRCNVSL-EDSVAELFDVDTS-QSGEYTCIVSNEA-GKASCTTHLY------
8794 ----PAKFVKRLND-----YSIEKGK----PLILEGTFT--GTPPISVTWKKN-G--------INVTP---S QRCNITT-TEKSAILEIPSSTVE-DAGQYNCYIENAS-GKDSCSAQIL------
8888 ----PPYFVKQLEPV----KVSV-GD----SASLQCQLA--GTPEIGVSWYKG-D--------TKLRP---T TTYKMHF-RNNVATLVFNQVDIN-DSGEYICKAENSV-GEVSASTFLT------
8984 ----PPSFSRQLRDV----QETV-GL----PVVFDCAIS--GSEPISVSWYKD-G--------KPLKD---S PNVQTSF-LDNTATLNIFKTDRS-LAGQYSCTATNPI-GSASSSARLILT----
9079 ----PPFFDIRLAP-----VDAVVGE----SADFECHVT--GTQPIKVSWAKD-S--------REIRS---G GKYQISY-LENSAHLTVLKVDKG-DSGQYTCYAVNEV-GKDSCTAQLN------
9176 ----PPSFTKRLSET----VEETEGN----SFKLEGRVA--GSQPITVAWYKN-N--------IEIQP---T SNCEITF-KNNTLVLQVRKAGMN-DAGLYTCKVSNDA-GSALCTSSIV------
9272 ----PPVFDQHLTP-----VTVSEGE----YVQLSCHVQ--GSEPIRIQWLKA-G--------REIKP---S DRCSFSF-ASGTAVLELRDVAIA-DSGDYVCKASNVA-GSDTTKSKVT------
9366 ----RLFFVSEPQS-----IRVVEKT----TATFIAKVG--GDPIPNVKWTK--G--------KWRQL-NQG GRVFIHQ-KGDEAKLEIRDTTKT-DSGLYRCVAFNEH-GEIESNVNLQ------
9581 ---EPVTLIKDIEN-----QTVLKDN----DAVFEIDIKINYPEIKLS-WYKGT---------EKL---EPS DKFEISI-DGDRHTLRVKNCQLK-DQGNYRLVCG-----PHIASAKLTVI----
9660 ----PAWERHLQD-----VTLKEGQ----TCTMTCQFSVPNVK-SE--WFRNG---------RILK---PQ GRHKTEV-EHKVHKLTIADVRAE-DQGQYTCKYE-----DLETSAELK------
9760 ----PIQFTKRIQN-----IVVSEHQ----SATFECEVSFDDAI-VT--WYKGP---------TELT---ES QKYNFRN-DGRCHYMTIHNVTPD-DEGVYSIVARLEPRGEARSTAELYLT----
12041 ----PLKFVKEIKD-----IILTESEFVGSSAIFECLVSPSTAI-TT--WMKDG---------SNIR---ES PKHRFIA-DGKDRKLHIIDVQLS-DAGEYTCVLRLGNK-EKTSTAKLV------
12138 ----PVRFVKTLEEE----VTVVKGQ----PLYLSCELN----KERDVVWFRKDGK--------IVVEK---P GRIVPGV-IGLMRALTINDADDT-DAGTYTVTVENAN-NLECS----------
12233 -----DWLVKPIRD-----QHVKPKG----TAIFACDIAKDTPNIK---WFKGY---------DEIPA-EPN DKTEILR-DGNHLYLKIKNAMPE-DIAEYAVEIE-----GKRYPAKLT------
12321 --EREVELLKPIED-----VTIYEKE----SASFDAEISEADIP-GQ--WKLKG---------ELLR---PS PTCEIKA-EGGKRFLTLRKVKLD-QAGEVLYQAL-----NAITTAILTVKEI--
12499 ---IRLKFMSPLED-----QTVKEGE----TATFVCELSHEKMH-VV--WLKND---------AKLH---TS RTVLISS-EGKTHKLEIMEVTLD-DISQIKAQVK-----ELSSTAQLK------
12590 -----PYFTVKLHD-----KTAVEKD----EITLKCEVSK-DVPVK---WFKDG---------EEIV---PS PKYSIKA-DGLRRILKIKKADLK-DKGEYVCDCG-----TDKTKANVT------
12674 -EARLIKVEKPLYG-----VEVFVGE----TAHFEIELSEPDVH-GQ--WKLKG---------QPLT---AS PDCEIIE-DGKKHILILHNCQLG-MTGEVSFQAA-----NAKSAANLKVKEL--
12766 ----PLIFITPLSD-----VKVFEKD----EAKFECEVSR-EPKTFR--WLKGT---------QEIT---GD DRFELIK-DGTKHSMVIKSAAFE-DEAKYMFEAE-----DKHTSGKLI------
12854 ---IRLKFLTPLKD-----VTAKEKE----SAVFTVELSHDNIR-VK--WFKND---------QRLH---TT RSVSMQD-EGKTHSITFKDLSID-DTSQIRVEAM-----GMSSEAKLTVLEG--
12945 -----PYFTGKLQD-----YTGVEKD----EVILQCEISKADAPVK---WFKDG---------KEIK---PS KNAVIKA-DGKKRMLILKKALKS-DIGQYTCDCG-----TDKTSGKLDIEDR--
13030 -EDREIKLVRPLHS-----VEVMETE----TARFETEISEDDIH-AN--WKLKG---------EALL---QT PDCEIKE-EGKIHSLVLHNCRLD-QTGGVDFQAA-----NVKSSAHLRVK----
13120 --PRVIGLLRPLKD-----VTVTAGE----TATFDCELSYEDIP-VE--WYLKG---------KKLE---PS DKVVPRS-EGKVHTLTLRDVKLE-DAGEVQLTAK-----DFKTHANLF------
13210 ---PPVEFTKPLED-----QTVEEGA----TAVLECEVSRENAK-VK--WFKNG---------TEIL---KS KKYEIVA-DGRVRKLVIHDCTPE-DIKTYTCDAK-----DFKTSCNLN------
13299 --PPHVEFLRPLTD-----LQVREKE----MARFECELSRENAK-VK--WFKDG---------AEIK---KG KKYDIIS-KGAVRILVINKCLLD-DEAEYSCEVR-----TARTSGMLT------
13388 ---EEAVFTKNLAN-----IEVSETD----TIKLVCEVSKPGAEVI---WYKGD---------EEII---ET GRYEILT-EGRKRILVIQNAHLE-DAGNYNCRLP-----SSRTDGKVKVHELA-
13479 -----AEFISKPQN-----LEILEGE----KAEFVCSISKESFPVQ---WKRDD---------KTLE---SG DKYDVIA-DGKKRVLVVKDATLQ-DMGTYVVMVG-----AARAAAHLT------
13565 ---EKLRIVVPLKD-----TRVKEQQ----EVVFNCEVN---TEGAKAKWFRNEE---------AIFD---S SKYIILQ-KDLVYTLRIRDADD-QANYNVSLTNHRGENVKSAANLI-------
13659 ---EDLRIVEPLKD-----IETMEKK----SVTFWCKVNRLNVT-LK--WTKNG---------EEVP---FD NRVSYRV-DKYKHMLTIKDCGFP-DEGEYIVTAG-----QDKSVAELLIIEA--
13749 ----PTEFVEHLED-----QTVTEFD----DAVFSCQLSREKAN-VK--WYRNG---------REIK---EG KKYKFEK-DGSIHRLIIKDCRLD-DECEYACGVE-----DRKSRARLF------
13837 ---IPVEIIRPPQD-----ILEAPGA----DVVFLAELNKDKVE-VQ--WLRNN---------MVVV---QG DHQMMS-EGKIHRLQICDIKPR-DQGEYRFIAK-----DKEARAKLELAAA--
13927 ----PKIKTADQD-----LVVDVGK----PLTMVVPYD--AYPKAEAEWFKENE---------PLS---- -TKTIDT-TAEQTSFRILEAKKG-DKGRYKIVLQNKH-GKAEGFINLK------
14319 ----EPTMDLSAFKD--G-LEVIVPN----PITILVP-ST-GYPRPTATWCFGDK---------VLE---TG DRVKMKT-LSAYAELVISPSERS-DKGIYTLKLENRV-KTISGEIDV-------
```

```
14615 -----PEIFLDVKLL--AGLTVKAGT----KIELPAT-VT-GKPEPKITWTKAD---------MILK---QD KRITIEN-VPKKSTVTIVDSKRS-DTGTYIIEAVNVC-GRATAVVEVNVL----
15314 -----PTIDLETHDI----IVIE-GE----KLSIPVP-FR-AVPVPTVSWHKDGK---------EVK---AS DRLTMKN-DHISAHLEVPKSVRA-DAGIYTITLENKL-GSATASINVK------
15608 -----PKVILRTSL------EVKRGD----EIALDASIS--GSPYPTITWIKDENVIVPEEIKKRAAPLVRR RKGEVQEEEPFVLPLTQRLSIDNSKKGESQLRVRDSL-RPDHGLYMIKVE----
16029 ----PPAVELDVSVK--GGIQIMAGK----TLRIPAV-VT-GRPVPTKVWTKEE---------GELD---KD -RVVIDN-VGTKSELIIKDALRK-DHGRYVITATNSC-GSKFAAARVE------
16322 ----PPTIKLRLSVRG-DTIKVKAGE----PVHIPADVT--GLPMPKIEWSKNET------VIEKPTDALQI TKEEVSR-SEAKTELSIPKAVRE-DKGTYTVTASNRL-GSVFRNVHVE------
16727 -----PEVFIDIGAQ--DCLVCKAGS----QIRIPAV-IK-GRPTPKSSWEFDGKAKK-AMKDGVHD---IP EDAQLET-AENSSVIIIPECKRS-HTGKYSITAKNKA-GQKTANCRVK------
17044 ----PPSIDLKEFME------VEEGT----NVNIVAK-IK-GVPFPTLTWFKAPPKKP-DNK-EPVL---YD THVNKLV-VDDTCTLVIPQSRRS-DTGLYTITAVNNL-GTASKEMRLN------
17449 -----PDLQLDASVR--DRIVVHAGG----VIRIIAY-VS-GKPPPTVTWNMN-----------ERT---LP QEATIET-TAISSSMVIKNCQRS-HQGVYSLLAKNEA-GERKKTII--------
17745 ----PPTLHLDFRDK----LTIRVGE----AFALTGR-YS-GKPKPKVSWFKDEA---------DVL---ED DRTHIKT-TPATLALEKIKAKRS-DSGKYCVVVENST-GSRKGFCQVN------
18143 ----PPELILDANMA--REQHIKVGD----TLRLSAI-IK-GVPFPKVTWKKED---------RD-----AP TKARIDV-TPVGSKLEIRNAAHE-DGGIYSLTVENPA-GSKTVS----------
18435 ----PPSVELDVKLI--EGLVVKAGT----TVRFPAI-IR-GVPVPTAKWTTDG---------SEIK---TD EHYTVET-DNFSSVLTIKNCLRR-DTGEYQITVSNAA-GSKTVAVHLT------
18833 ----PPEVELDVTCR--DVITVRVGQ----TIRILAR-VK-GRPEPDITWTKEG---------KVLV---RE KRVDLIQ-DLPRVELQIKEAVRA-DHGKYIISAKNSS-GHAQGSAIVN------
19128 -------PVLDLKLS--GVLTVKAGD----TIRLEAG-VR-GKPFPEVAWTKDKDA-------TDLT---RS PRVKIDT-RADSSKFSLTKAKRS-DGGKYVVTATNTA-GSFVAYATVN------
19531 ----PPEIDLDASMR--KLVIVRAGC----PIRLFAI-VR-GRPAPKVTWRKVG---------IDN----VV RKGQVDL-VDTMAFLVIPNSTRD-DSGKYSLTLVNPA-GEKAVF----------
19826 ----PPKILMPEQIT------IKAGK----KLRIEAH-VY-GKPHPTCKWKKGE---------DEVV---TS SHLAVHK-ADSSSILIIKDVTRK-DSGYYSLTAENSS-GTDTQKIKVV------
20220 ----PPRIDLSVAMK--SLLTVKAGT----NVCLDAT-VF-GKPMPTVSWKKDGT---------LLK---PA EGIKMAM-QRNLCTLELFSVNRK-DSGDYTITAENSS-GSKSATIKLK------
20618 ----PPEGELDADLR--KTLILRAGV----TMRLYVP-VK-GRPPPKITWSKPNV---------NLR---DR IGLDIKS-TDFDTFLRCENVNKY-DAGKYILTLENSC-GKKEYTIVVKV-----
20893 -----PDLDLKGLPD--LCYLAKENS----NFRLKIP-IK-GKPAPSVSWKKGED---------PLA---TD TRVSVES-SAVNTTLIVYDCQKS-DAGKYTITLKNVA-GTKEGTIS--------
21303 -----PTIVLDPTIK--DGLTIKAGD----TIVLNAISIL-GKPLPKSSWSKAG---------KDIR---PS DITQITS-TPTSSMLTIKYATRK-DAGEYTITATNPF-GTKVEHVKVT------
21701 -----PDFELDAELR--RTLVVRAGL----SIRIFVP-IK-GRPAPEVTWTKDNI---------NLK---NR A--NIEN-TESFTLLIIPECNRY-DTGKFVMTIENPA-GKKSGFVNVRVLD---
21990 -----PELDLRGIYQ--KLVIAKAGD----NIKVEIP-VL-GRPKPTVTWKKGDQ---------ILK---QT QRVNFET-TATSTILNINECVRS-DSGPYPLTARNIV-GEVGDVITIQVH----
22386 -----PKIKVDVKFK--DTVILKAGE----AFRLEAD-VS-GRPPPTMEWSKDGK--------ELEG---T- AKLEIKI-ADFSTNLVNKDSTRR-DSGAYTLTATNPG-GFAKHIFNVK------
22785 ----PPEIELDADLR--KVVTIRACC----TLRLFVP-IK-GRPAPEVKWARDHG---------ESL---DK A--SIES-TSSYTLLIVGNVNRF-DSGKYILTVENSS-GSKSAFVNVR------
23075 ----PP--AFKLLFN---TFTVLAGE----DLKVDVP-FI-GRPTPAVTWHKDNV---------PLK---QT TRVNAES-TENNSLLTIKDACRE-DVGHYVVKLTNSA-GEAIETLNVI------
23468 ----PPRISMDPKYK--DTIVVHAGE----SFKVDAD-IY-GKPIPTIQWIKGDQ--------ELSN---T- ARLEIKS-TDFATSLSVKDAVRV-DSGNYILKAKNVA-GERSVT----------
23867 -----PDIDLDLELR--KIINIRAGG----SLRLFVP-IK-GRPTPEVKWGKVDG---------EIR---DA A--IIDV-TSSFTSLVLDNVNRY-DSGKYTLTLENSS-GTKSAFVT--------
24157 -----P--DVKPAFS---SYSVQVGQ----DLKIEVP-IS-GRPKPTITWTKDGL---------PLK---QT TRINVTD-SLDLTTLSIKETHKD-DGGQYGITVANVV-GQKTAS----------
24550 -----PRISMDPKFR--DTIVVNAGE----TFRLEAD-VH-GKPLPTIEWLRGDK--------EIEE---S- ARCEIKN-TDFKALLIVKDAIRI-DGGQYILRASNVA-GSKSFPVNVK------
24949 -----PELDLDSELR--KGIVVRAGG----SARIHIP-FK-GRPTPEITWSREEG---------EFT---DK V--QIEK-GVNYTQLSIDNCDRN-DAGKYILKLENSS-GSKSAFVTVK------
25239 -----P--SLKLPFN---TYSIQAGE----DLKIEIP-VI-GRPRPNISWVKDGE---------PLK---QT TRVNVEE-TATSTVLHIKEGNKD-DFGKYTVTATNSA-GTATENLS--------
25632 -----PNASLDPKYK--DVIVVHAGE----TFVLEAD-IR-GKPIPDVVWSKDGK--------ELEE---TA ARMEIKS-TIQKTTLVVKDCIRT-DGGQYILKLSNVG-GTKSIPIT--------
26032 ----PPEIELDADLR--KVVVLRASA----TLRLFVT-IK-GRPEPEVKWEKAEG---------ILT---DR A--QIEV-TSSFTMLVIDNVTRF-DSGRYNLTLENNS-GSKTAFVNVR------
26322 -----P--SVELPFH---TFNVKARE----QLKIDVP-FK-GRPQATVNWRKDGQ---------TLK---ET TRVNVSS-SKTVTSLSIKEASKE-DVGTYELCVSNSA-GSITVPITII------
26714 ----PPRVMMDVKFR--DVIVVKAGE----VLKINAD-IA-GRPLPVISWAKDGI--------EIEE---R- ARTEIIS-TDNHTLLTVKDCIRR-DTGQYVLTLKNVA-GTRSVA----------
27101 -----PLFDIDSEMR--KTLIVKAGA----SFTMTVP-FR-GRPVPNVLWSKPDT---------DLR---TR A--YVDT-TDSRTSLTIENANRN-DSGKYTLTIQNVL-SAASLT----------
27797 -----PVIDLPLEYT--EVVKYRAGT----SVKLRAG-IS-GKPAPTIEWYKDDK---------ELQ---TN ALVCVEN-TTDLASILIKDADRL-NSGCYELKLRNAM-GSASATIRVQ------
28196 -----PEIDLDVALR--TSVIAKAGE----DVQVLIP-FK-GRPPPTVTWRKDEK---------NLG---SD ARYSIEN-TDSSSLLTIPQVTRN-DTGKYILTIENGV-GEP-KSSTVS------
28488 -----PEVDLSDIPG--AQVTVRIGH----NVHLELP-YK-GKPKPSISWLKDGL---------PLK---ES EFVRFSK-TENKITLSIKNAKKE-HGGKYTVILDNAV-CRIAVPIT--------
28882 ----PPIVEFGPEYF--DGLIIKSGE----SLRIKAL-VQ-GRPVPRVTWFKDGV--------EIEK---R- MNMEITD-VLGSTSLFVRDATRD-HRGVYTVEAKNAS-GSAKAEIKVK------
29282 -----PELDIDANFK--QTHVVRAGA----SIRLFIA-YQ-GRPTPTAVWSKPDS---------NLS---LR A--DIHT-TDSFSTLTVENCNRN-DAGKYTLTVENNS-GSKSIT----------
29568 -PQIEPTADLTGITN--QLITCKAGS----PFTIDVP-IS-GRPAPKVTWKLEEM---------RLK---ET DRVSITT-TKDRTTLTVKDSMRG-DSGRYFLTLENTA-GVKTFSVTVV------
29971 ----PPKAELDARLHG-DLVTIRAGS----DLVLDAA-VG-GKPEPKIIWTKGD---------KELD---LC EKVSLQY-TGKRATAVIKFCDRS-DSGKYTLTVKNAS-GTKAVS----------
30372 -----PDLELADDLK--KTVTIRAGA----SLRLMVS-VS-GRPPPVITWSKQGI---------DLA---SR A--IIDT-TESYSLLIVDKVNRY-DAGKYTIEAENQS-GKKSATVLVK------
30663 -----PTIDLSTMPQ--KTIHVPAGR----PVELVIP-IA-GRPPPAASWFFAGS---------KLR---ES ERVTVET-HTKVAKLTIRETTIR-DTGEYTLELKNVT-GTTSETIKVI------
31061 -----PDYELDERYQ--EGIFVRQGG----VIRLTIP-IK-GKPFPICKWTKEG---------QDIS---KR --AMIAT-SETHTELVIKEADRG-DSGTYDLVLENKC-GKKAVYIKVR------
31460 -----PGIRKEMKD-----VTTKLGE----AAQLSCQIV--GRPLPDIKWYRF-G--------KELIQ-SRK YKMSSDG--RT-HTLTVMTEEQE-DEGVYTCIATNEV-GEVETSSKLL------
31553 -----PQFHPGYPLK--EKYYGAVGS----TLRLHVM-YI-GRPVPAMTWFHGQK--------LLQN---S- ENITIEN-TEHYTHLVMKNVQRKTHAGKYKVQLSNVF-GTVDAILDVEI-----
31855 -----PHFKEELRN-----LNVRYQS----NATLVCKVT--GHPKPIVKWYRQ-G--------KEIIA-DGL KYRIQEF-KGGYHQLIIASVTDD-DATVYQVRATNQG-GSVSGTASLE------
31955 ----------PKTLEGMGAVHALRGE----VVSIKIP-FS-GKPDPVITWQKGQD---------LID---NN GHYQVIV-TRSFTSLVFPNGVERKDAGFYVVCAKNRF-GIDQKTVELDVA----
32496 -----PVSGQIMHA------VGEEGG----HVKYVCKIEN-YDQSTQVTWYFGVR---------QLEN---S EKYEITY-EDGVAILYVKDITKL-DDGTYRCKVVNDY-GEDSSYAELF------
32617 ----PPEFTLPLYN-----KTAYVGE----NVRFGVTIT--VHPEPHVTWYKS-G--------QKIKPGDND KKYTFES-DKGLYQLTINSVTTD-DDAEYTVVARNKY-GEDSCKAKLTVT----
32722 -----PMFKRLLAN-----AECQEGQ----SVCFEIRVS--GIPPPTLKWEKD-G--------QPLSL-GP- NIEIIHE-GLDYYALHIRDTLPE-DTGYYRVTATNTA-GSTSCQAHLQ------
33301 -----PRITLRMRS-----HRVPCGQ----NTRFILNVQ--SKPTAEVKWYHN-G--------VELQE-SS- KIHYTN--TSGVLTLEILDCHTD-DSGTYRAVCTNYK-GEASDYATLDVT----
33488 -----ARILTKPRS-----MTVYEGE----SARFSCDTD--GEPVPTVTWLRK-G--------QVLST-SAR HQVTT---TKYKSTFEISSVQAS-DEGNYSVVVENSE-GKQEAEFTLT------
33645 ----PPKITQFLKA-------EASKE----IAKLTCVVESSVLRAKEVTWYKDGK---------KLKE---N GHFQFHYSADGTYELKINNLTES-DQGEYVCEISGEG-GTSKTNLQ--------
33779 -----PVIVTGLQD-----TTVSSDS----VAKFAVKAT--GEPRPTAIWTKD-G--------KAITQ-GG- -KYKLSE-DKGGFFLEIHKTDTS-DSGLYTCTVKNSA-GSVSSSCKLT------
33963 ---RTHAEIKAFSTQ----MSINEGQ----RLVLKANIA--GATDVK--WVLN-G--------VELTN---S EEYRYGV-SGSDQTLTIKQASHR-DEGILTCISKTKE-GIVKCQYDLT------
34061 -----PAFISQPRS-----QNINEGQ----NVLFTCEIS--GEPSPEIEWFKN-N--------LPISI---S SNVSISR-SRNVYSLEIRNASVS-DSGKYTIKAKNFR-GQCSATASLM------
```

Multiple alignment of the 163 Ig domains in the canonical titin (Q8WZ42-1) including extra 12 Ig domains manually identified by us (see methods). The position of the first residue is indicated for each Ig domain (first column). Manually annotated domains are shown in red. Cysteines are highlighted in yellow. The 3 highly conserved cysteines are indicated on top of the alignment following I91's numbering (pdb: 1TIT, see supplementary text 4). The I65-I70 segment (6 domains, pdb: 3B43, starting in amino acid 7946) and I91 (pdb: 1TIT, starting in amino acid 12674) are indicated in blue.

Supplementary Note 4 – Sequence reference of I91

For clarity and consistency with previous studies [2,9], we refer to the amino acids in the titin Ig sequence alignment according to the numbering of the human titin domain I91 (pdb: 1TIT, also known as I27). In particular, the three conserved cysteines (Supplementary Note 2) observed in titin's Igs are aligned with residues I23, F73 and S80 of I91 (see sequence below). We verified that the alignment of these three residues, which all appear in well-conserved regions, is robust and unaffected by the chosen alignment program (ClustalW, Muscle, Tcoffee). The sequence of I91 (as it appears in pdb 1TIT) together with reference numbering is indicated below.

L$_{1}$IEVEKPLYG$_{10}$VEVFVGETAH$_{20}$FEI$_{23}$ELSEPDV$_{30}$HGQWKLKGQP$_{40}$LTASPDCEII$_{50}$EDGKKHIL$_{60}$ILHN
CQLGMTGE$_{70}$VSF$_{73}$QAANAKS$_{80}$AANLKVKEL$_{89}$

Supplementary Note 5 – Search of the cysteine triad in protein domains

We inspected a comprehensive database of protein structures in order to identify cysteine triads similar to the ones observed in titin. We used the pdb database, NCBI non-redundant subset nrprot (01.16.13 version) with a p-value threshold of 10^{-7}, which guarantees a comprehensive coverage of protein diversity and a reasonable number of proteins to analyze (12382 pdb files). We wrote a Python2.7 script to identify motifs of three or more clustered cysteines. For this automatic research, we considered that 3 cysteines are clustered if their Cβ are located within a sphere of diameter inferior to 6 Å. With this definition of clustered cysteines, both titin I67 and I69 from the crystallographic structure of I65-I70 (pdb:3B43) display a motif of 3 co-localized cysteines. We screened the 12387 representative structures and retrieved 952 structures with 3 (69%), 4 (27%), 5(4%) or 6(0.02%) clustered cysteines. The major part of the clustered cysteines participates in a metal binding site. We can track the presence of metal sites by searching the keyword "METAL" in the pdb header. In this way, we removed 602 proteins known to bind metals. Then, we inspected each of the remaining 350 proteins with the visualization program Pymol. The majority of these 350 pdb structures are cysteine-rich proteins marked by marginal secondary structures (such as toxins) and 2 or more vicinal disulfide bonds (an even number of cysteines are observed in these structures). In 24 proteins, we found an odd number of clustered cysteines, but in all of them, the cysteines emerge in unstructured regions (absence of secondary structures) with the thiol group largely exposed to the solvent. In conclusion, we could not identify other proteins with a structural motif composed of a cysteine triad, buried in a stable hydrophobic core, as observed in the X-ray structure of I67 and I69. Even though disulfide bonds are abundant in immunoglobulin domains, the presence of an odd number of clustered cysteine seems quite specific to the Ig modules of titin. A potential limitation of our analysis is that it only considers available high-resolution structures. Similar cysteine triads may be found in Ig domains for which there is no high-resolution structure. Indeed, the triad-forming cysteines are present in domain C10 of the cardiac myosin binding protein C (Uniprot: Q14896), a sarcomeric protein that modulates actomyosin contraction [10].

Supplementary Note 6 – The proportion of disulfide-containing Ig domains changes in different isoforms of titin

We study the presence of disulfide-forming CysB, CysF and CysG in the I-band Ig domains of five representative cardiac and skeletal titin isoforms available in Uniprot (Q8WZ42). Supplementary Table 5 shows how alternative splicing critically modulates the length of the isoforms and sets the content of cysteines B, F and G. The I65-I70 fragment is present in the isoforms N2BA, Soleus and Novex-2 and absent from the N2B and Novex-3 isoforms.

Supplementary Note 7 – Protein sequences

Proteins were cloned in pQE80L vector from Qiagen. Sequences in bold correspond to the initial methionine, a histidine tag, pairs of amino acids resulting from cloning sites, and two terminal cysteines.

I65-70

MRGSHHHHHHGSGAMEPPYFIEPLEHVEAAIGEPITLQCKVDGTPEIRIAWYKEHTKLRSAPAYKMQ
FKNNVASLVINKVDHSDVGEYTCKAENSVGAVASSAVLVIKERKLPPSFARKLKDVHETLGFPVAFECRI
NGSEPLQVSWYKDGELLKDDANLQTSFIHNVATLQILQTDQSHVGQYNCSASNPLGTASSSAKLTLSEHE
VPPFFDLKPVSVDLALGESGTFKCHVTGTAPIKITWAKDNREIRPGGNYKMTLVENTATLTVLKVTKGD
AGQYTCYASNVAGKDSCSAQLGVQEPPRFIKKLEPSRIVKQDEHTRYECKIGGSPEIKVLWYKDETEIQES
SKFRMSFVESVAVLEMYNLSVEDSGDYTCEAHNAAGSASSSTSLKVKEPPVFRKKPHPVETLKGADVHL
ECELQGTPPFQVSWHKDKRELRSGKKYKIMSENFLTSIHILNVDSADIGEYQCKASNDVGSDTCVGSITLK
APPRFVKKLSDISTVVGEEVQLQATIEGAEPISVAWFKDKGEIVRESDNIWISYSENIATLQFSRAEPANA
GKYTCQIKNEAGTQECFATLSVLE**RSCC**

(I69)$_8$

MRGSHHHHHHGSVKEPPVFRKKPHPVETLKGADVHLECELQGTPPFQVSWHKDKRELRSGKKYKI
MSENFLTSIHILNVDSADIGEYQCKASNDVGSDTCVGSITLK**RS**VKEPPVFRKKPHPVETLKGADVHLEC
ELQGTPPFQVSWHKDKRELRSGKKYKIMSENFLTSIHILNVDSADIGEYQCKASNDVGSDTCVGSITLK**R**
SVKEPPVFRKKPHPVETLKGADVHLECELQGTPPFQVSWHKDKRELRSGKKYKIMSENFLTSIHILNVD
SADIGEYQCKASNDVGSDTCVGSITLK**RS**VKEPPVFRKKPHPVETLKGADVHLECELQGTPPFQVSWHK
DKRELRSGKKYKIMSENFLTSIHILNVDSADIGEYQCKASNDVGSDTCVGSITLK**RS**VKEPPVFRKKPHPV
ETLKGADVHLECELQGTPPFQVSWHKDKRELRSGKKYKIMSENFLTSIHILNVDSADIGEYQCKASNDVG
SDTCVGSITLK**RS**VKEPPVFRKKPHPVETLKGADVHLECELQGTPPFQVSWHKDKRELRSGKKYKIMSE
NFLTSIHILNVDSADIGEYQCKASNDVGSDTCVGSITLK**RS**VKEPPVFRKKPHPVETLKGADVHLECELQ
GTPPFQVSWHKDKRELRSGKKYKIMSENFLTSIHILNVDSADIGEYQCKASNDVGSDTCVGSITLK**RS**VK
EPPVFRKKPHPVETLKGADVHLECELQGTPPFQVSWHKDKRELRSGKKYKIMSENFLTSIHILNVDSADI
GEYQCKASNDVGSDTCVGSITLK**RSCC**

(I91ΔCys)$_2$-I69-(I91ΔCys)$_2$

MRGSHHHHHHGSLIEVEKPLYGVEVFVGETAHFEIELSEPDVHGQWKLKGQPLAASPDAEIIEDGKK
HILILHNAQLGMTGEVSFQAANTKSAANLKVKEL**RS**LIEVEKPLYGVEVFVGETAHFEIELSEPDVHGQW
KLKGQPLAASPDAEIIEDGKKHILILHNAQLGMTGEVSFQAANTKSAANLKVKEL**RS**VKEPPVFRKKPHP
VETLKGADVHLECELQGTPPFQVSWHKDKRELRSGKKYKIMSENFLTSIHILNVDSADIGEYQCKASNDV
GSDTCVGSITLK**RS**LIEVEKPLYGVEVFVGETAHFEIELSEPDVHGQWKLKGQPLAASPDAEIIEDGKKHI
LILHNAQLGMTGEVSFQAANTKSAANLKVKEL**RS**LIEVEKPLYGVEVFVGETAHFEIELSEPDVHGQWK
LKGQPLAASPDAEIIEDGKKHILILHNAQLGMTGEVSFQAANTKSAANLKVKEL**RSCC**

Supplementary references

1 Lu, H. & Schulten, K. Steered molecular dynamics simulations of force-induced protein domain unfolding. *Proteins* **35**, 453-463 (1999).

2 Marszalek, P. E. *et al.* Mechanical unfolding intermediates in titin modules. *Nature* **402**, 100-103, doi:10.1038/47083 (1999).

3 Ainavarapu, S. R. *et al.* Contour length and refolding rate of a small protein controlled by engineered disulfide bonds. *Biophys J* **92**, 225-233, doi:10.1529/biophysj.106.091561 (2007).

4 Watanabe, K., Muhle-Goll, C., Kellermayer, M. S., Labeit, S. & Granzier, H. Different molecular mechanics displayed by titin's constitutively and differentially expressed tandem Ig segments. *Journal of structural biology* **137**, 248-258, doi:10.1006/jsbi.2002.4458 (2002).

5 Alegre-Cebollada, J., Kosuri, P., Rivas-Pardo, J. A. & Fernandez, J. M. Direct observation of disulfide isomerization in a single protein. *Nature chemistry* **3**, 882-887, doi:10.1038/nchem.1155 (2011).

6 Bustamante, C., Marko, J. F., Siggia, E. D. & Smith, S. Entropic elasticity of lambda-phage DNA. *Science* **265**, 1599-1600 (1994).

7 Carrion-Vazquez, M., Marszalek, P. E., Oberhauser, A. F. & Fernandez, J. M. Atomic force microscopy captures length phenotypes in single proteins. *Proceedings of the National Academy of Sciences of the United States of America* **96**, 11288-11292 (1999).

8 Dougan, L., Li, J., Badilla, C. L., Berne, B. J. & Fernandez, J. M. Single homopolypeptide chains collapse into mechanically rigid conformations. *Proceedings of the National Academy of Sciences of the United States of America* **106**, 12605-12610, doi:0900678106 [pii]
10.1073/pnas.0900678106 (2009).

9 Li, H. *et al.* Reverse engineering of the giant muscle protein titin. *Nature* **418**, 998-1002, doi:10.1038/nature00938 (2002).

10 Bennett, P. M., Furst, D. O. & Gautel, M. The C-protein (myosin binding protein C) family: regulators of contraction and sarcomere formation? *Rev Physiol Biochem Pharmacol* **138**, 203-234 (1999).

11 Li, H. & Fernandez, J. M. Mechanical design of the first proximal Ig domain of human cardiac titin revealed by single molecule force spectroscopy. *Journal of molecular biology* **334**, 75-86 (2003).

12 Alegre-Cebollada, J. *et al.* S-glutathionylation of cryptic cysteines enhances titin elasticity by blocking protein folding. *Cell* **156**, 1235-1246, doi:10.1016/j.cell.2014.01.056 (2014).

13 Bell, G. I. Models for the specific adhesion of cells to cells. *Science* **200**, 618-627 (1978).

14 Kosuri, P. *et al.* Protein folding drives disulfide formation. *Cell* **151**, 794-806, doi:10.1016/j.cell.2012.09.036 (2012).

15 Koti Ainavarapu, S. R., Wiita, A. P., Dougan, L., Uggerud, E. & Fernandez, J. M. Single-molecule force spectroscopy measurements of bond elongation during a bimolecular reaction. *Journal of the American Chemical Society* **130**, 6479-6487, doi:10.1021/ja800180u (2008).

16 Wiita, A. P. *et al.* Probing the chemistry of thioredoxin catalysis with force. *Nature* **450**, 124-127, doi:10.1038/nature06231 (2007).

17 Popa, I., Kosuri, P., Alegre-Cebollada, J., Garcia-Manyes, S. & Fernandez, J. M. Force dependency of biochemical reactions measured by single-molecule force-clamp spectroscopy. *Nature protocols* **8**, 1261-1276, doi:10.1038/nprot.2013.056 (2013).

18 Schlierf, M., Li, H. & Fernandez, J. M. The unfolding kinetics of ubiquitin captured with single-molecule force-clamp techniques. *Proceedings of the National Academy of Sciences of the United States of America* **101**, 7299-7304, doi:10.1073/pnas.0400033101 (2004).

19 Fernandez, J. M. & Li, H. Force-clamp spectroscopy monitors the folding trajectory of a single protein. *Science* **303**, 1674-1678, doi:10.1126/science.1092497 (2004).

20 Bang, M. L. *et al.* The complete gene sequence of titin, expression of an unusual approximately 700-kDa titin isoform, and its interaction with obscurin identify a novel Z-line to I-band linking system. *Circulation research* **89**, 1065-1072 (2001).

21 Granzier, H. L. & Labeit, S. The giant protein titin: a major player in myocardial mechanics, signaling, and disease. *Circulation research* **94**, 284-295, doi:10.1161/01.RES.0000117769.88862.F8 (2004).

22 Kellermayer, M. S. Z. & Grama, L. Stretching and visualizing titin molecules: combining structure, dynamics and mechanics. *Journal of muscle research and cell motility* **23**, 499-511, doi:Doi 10.1023/A:1023414624092 (2002).

23 Miseta, A. & Csutora, P. Relationship between the occurrence of cysteine in proteins and the complexity of organisms. *Molecular biology and evolution* **17**, 1232-1239 (2000).

24 Hazes, B. & Dijkstra, B. W. Model building of disulfide bonds in proteins with known three-dimensional structure. *Protein engineering* **2**, 119-125 (1988).

25 Letunic, I. & Bork, P. Interactive Tree Of Life (iTOL): an online tool for phylogenetic tree display and annotation. *Bioinformatics* **23**, 127-128, doi:10.1093/bioinformatics/btl529 (2007).

26 Kruger, M. *et al.* Protein kinase G modulates human myocardial passive stiffness by phosphorylation of the titin springs. *Circulation research* **104**, 87-94, doi:10.1161/CIRCRESAHA.108.184408 (2009).

27 Hidalgo, C. *et al.* PKC phosphorylation of titin's PEVK element: a novel and conserved pathway for modulating myocardial stiffness. *Circulation research* **105**, 631-638, 617 p following 638, doi:10.1161/CIRCRESAHA.109.198465 (2009).
